# Periodontal Inflammation and Systemic Diseases: An Overview

**DOI:** 10.3389/fphys.2021.709438

**Published:** 2021-10-27

**Authors:** Mireya Martínez-García, Enrique Hernández-Lemus

**Affiliations:** ^1^Sociomedical Research Unit, National Institute of Cardiology “Ignacio Chávez”, Mexico City, Mexico; ^2^Computational Genomics Division, National Institute of Genomic Medicine (INMEGEN), Mexico City, Mexico; ^3^Centro de Ciencias de la Complejidad, Universidad Nacional Autónoma de Mèxico, Mexico City, Mexico

**Keywords:** periodontitis, systemic inflammation, systemic diseases, molecular mechanisms, chronic inflammation, cytokines, oral pathogens

## Abstract

Periodontitis is a common inflammatory disease of infectious origins that often evolves into a chronic condition. Aside from its importance as a stomatologic ailment, chronic periodontitis has gained relevance since it has been shown that it can develop into a systemic condition characterized by unresolved hyper-inflammation, disruption of the innate and adaptive immune system, dysbiosis of the oral, gut and other location's microbiota and other system-wide alterations that may cause, coexist or aggravate other health issues associated to elevated morbi-mortality. The relationships between the infectious, immune, inflammatory, and systemic features of periodontitis and its many related diseases are far from being fully understood and are indeed still debated. However, to date, a large body of evidence on the different biological, clinical, and policy-enabling sources of information, is available. The aim of the present work is to summarize many of these sources of information and contextualize them under a systemic inflammation framework that may set the basis to an integral vision, useful for basic, clinical, and therapeutic goals.

## Introduction

Periodontal disease (PD) is a general term encompassing a group of inflammatory pathologies that mainly include gingivitis and periodontitis. It is particularly pervasive in adults (Bui et al., 2019; de Molon et al., 2019), though, is not uncommon in children (Alrayyes and Hart, 2011; Reis et al., 2021). Indeed, PD is an often *all-encompassing*-term used to refer to any of the wide spectrum of inflammatory diseases able to affect the periodontium. The periodontium being itself an umbrella term, that comprises a number of different structures supporting the teeth: gingiva, cementum, periodontal ligament, and alveolar bone (Kinane et al., 2017). PD is often initiated by an uncontrolled inflammatory response to a slow and constant bacterial colonization of the tooth surface and soft gingival tissues—Gingivitis (Graves, 2008), but it is the host inflammatory response to the microbial challenge that is responsible for the degradation of the periodontium—i.e., Periodontitis (Balta et al., 2017).

During periodontitis, pathogens trigger the leukocytes of the innate immune system to release pro-inflammatory mediators, such as cytokines, that play an essential role in the progression of chronic periodontitis (CP) (Ramadan et al., 2020). Although infection is a necessary condition for the development of PD and CP, as far as it is known, infection alone is not sufficient for disease progression. The pathogens activate the acquired immune system contributing to an exacerbated progression of the inflammatory condition. As the immune response continues, serious damage occurs to both soft and hard periodontal tissues (Ramadan et al., 2020). For instance, it has been argued that individual susceptibility purely associated to organismal immune and inflammatory responses is also essential (Cecoro et al., 2020). This is so, since most signaling pathways and even cellular and tissular events common to these disorders have been traced to a set of concurrent molecular origins (Kinane et al., 2017).

The association between oral inflammation in PD and systemic inflammation is essential to understand the long term detrimental effects that periodontal inflammation may have on the systemic behavior of a multitude of organs. This may lead to unveil the extent to which oral disease may raise the risk of developing non-oral conditions (Bui et al., 2019; Konkel et al., 2019). In this review article, we will summarize some recent advances and the state of the art in our understanding of the relation between periodontitis and its associated systemic inflammation with a number of biological phenomena at the molecular and functional level. We will also discuss its genetic origins and analyze the known associations of periodontal inflammation with other chronic diseases, as well as some prognostic, diagnostic, and therapeutic tools related to these associations in order to sketch the full spectrum of influence of PD in human health.

## 1. Biological Aspects of Periodontal Inflammation

**Figure 1:**
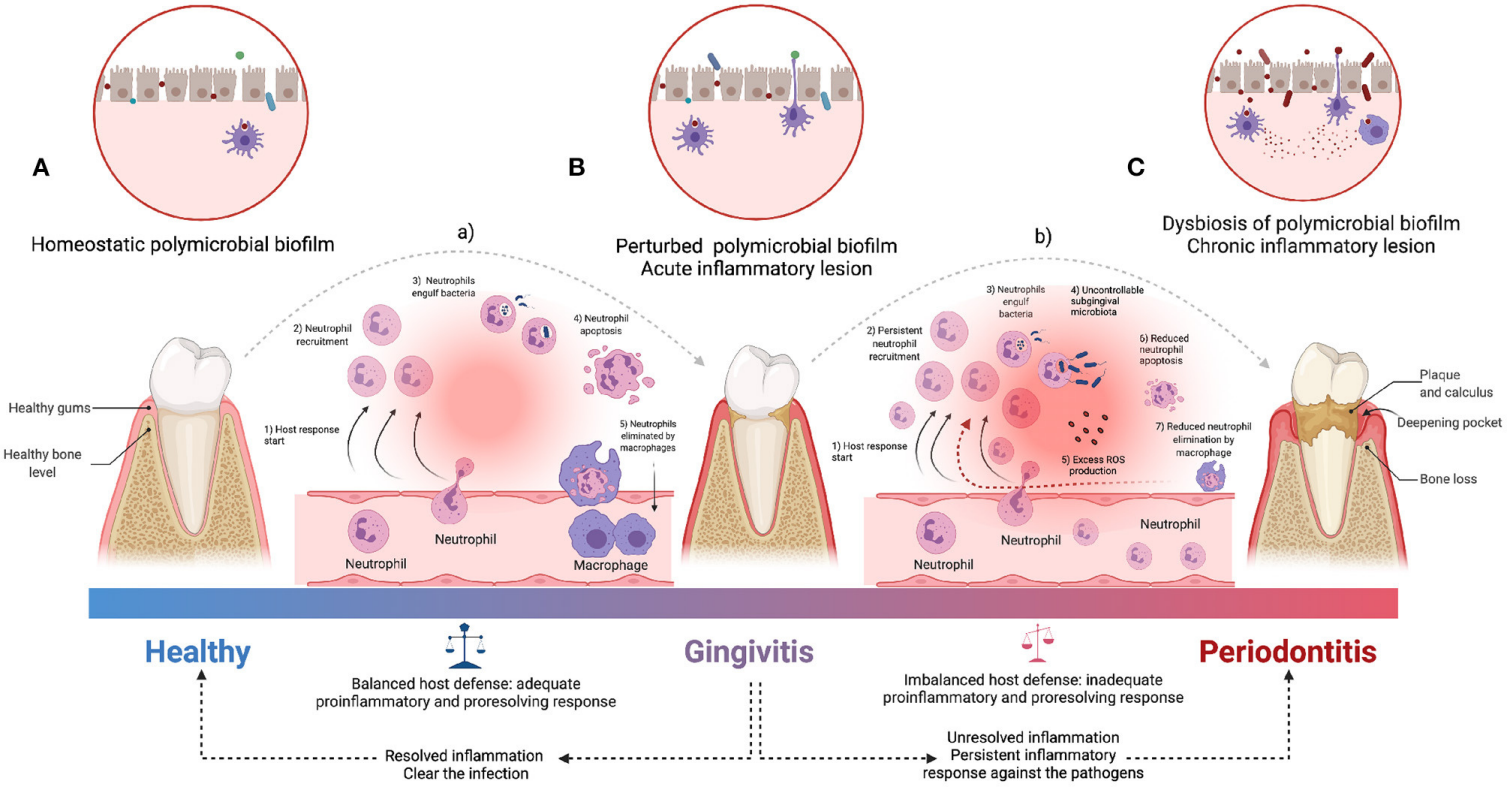
An overview of the pathogenesis of periodontitis. The acute inflammatory process is started by the infiltration of leukocytes (a), which limit bacterial invasion of **(A)** homeostatic state to **(B)** perturbed state (of healthy conditions to gingivitis stage). The pro-resolution mediators downregulate the recruitment of immune cells and uptake of apoptotic neutrophils by macrophages to facilitate clearance of the inflammatory lesion (Adequate balance host defense). Periodontitis is characterized by both **(C)** dysbiosis of oral microbiota and persistent pro-inflammatory state derived from the inability to clear the infection (b), followed by unresolved lesion leading to immune cell-mediated self-destruction of periodontal tissues which results in chronic pathology (Inadequate balance host defense). ROS, Reactive oxygen species [Figure generated with BioRender (Biorender.com)].

PD and CP are complex organismal conditions whose underlying biology is yet to be fully discerned. However, important advances have been made in our understanding of the fundamental molecular and physiological mechanisms behind them. The first section of this review will be devoted to discuss recent works centered on the biological features of periodontal disease. The greatest impacts of PD and CP in overall health are arguably given via the induction of systemic inflammation (Chi et al., 2010; Hirschfeld et al., 2019). Its origins have been traced back to the oral microbiota, immune system responses, and genetic constitution of the host (Figure 1) (Kinane et al., 2017). However, due to the complexity of the responses and the heterogeneity of the evidence, the actual roles, and mechanistic details of the relationship between periodontitis and systemic diseases are still being debated (Saranyan et al., 2017; Ide, 2021).

Of particular interest is the physiology of the individual's immune response. The presence of certain periodontal microorganisms initiates immune responses (Bartold and Van Dyke, 2019; Güler et al., 2020) involving both innate immunity players [such as macrophages, dendritic cells, natural killer (NK) cells, monocytes, neutrophils] as well as adaptive immunity (represented by the concerted action of B and T lymphocytes) resulting in the production and release of pro-inflammatory molecules ([such as interferon (IFN)-γ, interleukin (IL)-17, tumor necrosis factor (TNF), IL-1, IL-6] and associated enzymes—in particular collagenases such as matrix metalloproteinases (MMPs) (Figures 1A,B) (Franco et al., 2017)—(Holmström et al., 2017; Nascimento et al., 2017; Dahlen et al., 2019; Cecoro et al., 2020).

In this regard, it is interesting to notice that inflammation here plays a dual role: Inflammatory response is a physiological reaction aimed at protecting the organism against bacterial infections reaching *deeper* tissues (such as bones) (Könönen et al., 2019; Pan et al., 2019; Ramadan et al., 2020). However, when inflammation becomes deregulated and persistent (Figures 1B,C), it may lead to an irreversible destruction of the periodontal tissues, thus leading to periodontitis and its symptomatic consequences such as periodontal pockets, attachment loss, gingival recessions, tooth mobility, tooth migration, and tooth loss (Figure 1C) (Scannapieco, 2004; Offenbacher et al., 2008; Garlet, 2010; Hernández-Monjaraz et al., 2018). This way, the dentogingival epithelial surface area—which may include pocket epithelium adjacent to subgingival biofilm—becomes the battleground allowing local inflammation to disrupt systemic health (Nascimento et al., 2017; Kurgan and Kantarci, 2018; Cecoro et al., 2020).

Dysruption of local inflammation has a number of disease-related outcomes (Valentine et al., 2016). This may happen via periodontitis-associated inflammatory cascades: locally produced pro-inflammatory mediators enter into systemic circulation, thus becoming able to affect distant organs disrupting inflammatory state equilibria (Hajishengallis, 2015). Indeed, when contrasted with healthy subjects, patients with periodontitis present higher values of circulating white blood cells as well as other systemic inflammatory parameters such as C-reactive protein (CRP), a protein produced by the liver as a response to external stress. Hence, we can argue that periodontitis-associated local inflammation may become systemic, thus modifying organismal inflammatory loads and conversely, systemic inflammation alters periodontal health (Cecoro et al., 2020). In this regard, D'aiuto and colleagues have explored the relationship between oxidative stress processes and systemic inflammation in the context of severe and chronic PD (Daiuto et al., 2010; Li et al., 2019b).

Oxidative stress and molecular pathways of inflammation have also been discussed in connection with the relationship between myocardial infarction and periodontitis (Górski et al., 2016; Sarda et al., 2016; D́ıaz et al., 2020). The role of oxidative stress is further stressed by the finding that SOD2 (an important antioxidant enzyme) is upregulated in periodontitis, likely as an equilibrating response to inflammatory progression (Yamamoto et al., 2016; Yoon et al., 2018).

Along these lines, it has been noted that inflammation in most common chronic diseases such as diabetes, obesity, cardiovascular and neurological diseases, etc., involves low-grade inflammation (LGI). In some cases LGI has been linked to PD. Causality, however, may not be so established (Moutsopoulos and Madianos, 2006). Hence, it has been hypothesized that periodontal inflammation is causally linked to the development and progression of chronic systemic diseases by a mechanism of LGI induction, which is considered as *a silent risk factor* for many of them. LGI is, however, elusive: CRP values above 3 mg/L, but below 10 mg/L, have been reported as being indicative of LGI. Caution must be taken, since CRP is a non-specific inflammatory marker and its values can suddenly vary based on multiple causes (Loos, 2005; Cecoro et al., 2020). Some authors have indeed argued that LGI may account for the association between periodontitis and other systemic comorbidities, though this is still being debated, as are indeed many of the subtler connections between periodontal inflammation and systemic diseases (Hoare et al., 2019; Cecoro et al., 2020).

In this regard, we can notice that there is clinical evidence implicating periodontitis in the pathogenesis of systemic inflammation. However, a cause-and-effect relationship is yet to be demonstrated (Ide, 2021). Some studies shown a decrease in systemic inflammatory biomarkers following periodontal interventions and consequent possible benefit in the reduction of cardiovascular risk as endothelial dysfunction. However, evidence of its effects on cardiovascular events in the long term is still limited and there is no evidence that they prevent atherosclerotic vascular disease or modify its outcomes, so further studies are needed (Ferlazzo et al., 2021). Also, periodontal treatment has been shown to be associated with a modest reduction of glycated hemoglobin (HbA1c) in subjects with type 2 diabetes. However, there is limited confidence in the conclusion due to a lack of multicenter trials of sufficient sample size (Li et al., 2020; Preshaw et al., 2020). Likewise, it has not been possible to determine whether there is a benefit of periodontal treatment in women at risk of obstetric complications, however, it seems to be a good practice to apply preventive strategies in women with periodontitis (Mohr et al., 2019; Ye et al., 2020).

### 1.1. Pathogenesis of Periodontal Inflammation

Molecular and physiological changes leading to PD originate in microbial dysbiosis (i.e., a disruption in the microbiota homeostasis due to an imbalance in the functional composition and metabolic activities of the microbial species, see Figures 1A,B) in the oral cavities. A dysbiotic microbiome—mainly Gram-negative anaerobic bacteria—, established either in the enamel tooth surfaces or below the gingival margin may trigger innate immunity pathways by chemical stimulation of neighboring cells in the periodontal epithelium (Kumar, 2017; Fujita et al., 2018, see Figure 1). This may also stimulate the periodontal ligament and gingival fibroblasts, as well as dendritic cells releasing mediators of inflammation [via toll-like receptors (TLRs)] in response to bacterial endotoxins (Song et al., 2017; Behm et al., 2019). Neighboring cells located in the connective tissue and the alveolar bone drive the expression of pro-inflammatory cytokines and chemokines, including TNF-α, IL-1β, IL-6, IL-8, IL-12, IL- 17, and the receptor activator of the nuclear factor kappa B ligand (RANK-L) (Duka et al., 2019).

Failure of infection resolution sustains the release of pro-inflammatory mediators triggering adaptive immunity through the respective activation of B and T cells. Once connective tissue is infiltrated by lymphocytes with more RANK-L+ B cells than T cells, the latter will produce TNF-α that in conjunction with RANK-L and IL-17 will increase osteoclastogenesis, bone resorption and clinical attachment loss (CAL) (Bostanci et al., 2019; de Molon et al., 2019). This is just the start of inflammatory delocalization (Hasturk and Kantarci, 2015; Loos and Van Dyke, 2020).

The pathogenic processes may also be able to extend to other anatomic locations leading to systemic inflammation. Periodontal pathogens may be able indeed to promote development of non-oral diseases either indirectly—as we just described—or directly. Oral microbial dysbiosis may directly induce systemic inflammation, either by increasing inflammation by firsthand toxin release or by the transport of microbial products into the bloodstream (Bui et al., 2019).

Among the abundant microbial species we may include bacteria from the following families: *Treponema, Bacteroides, Porphyromonas, Prevotella, Capnocytophaga, Peptostreptococcus, Fusobacterium, Actinobacillus*, and *Eikenella* (Bui et al., 2019). To date, at least three microbial species have been demonstrated to be strongly associated with PD: *Porphyromonas gingivalis, Treponema denticola*, and *Tannerella forsythia*. These bacteria, often present in the form of a microbial community, are often called the *red complex*. These are not, however, the only oral microbes that may exert some effect in the oral microbial niche (Bui et al., 2019). Other oral pathogens such as *Entamoeba gingivalis* have recently been revealed as relevant players in the onset and development of PD (Bonner et al., 2018).

Recent studies have argued that the oral cavity contains between 500 and 700 prevalent taxa; conforming what we know as the oral microbiota (or oral microbiome), which is present either in saliva, gingival epithelium, and other inner surfaces of the oral cavity; particularly concentrated in the dental plaque (Diomede et al., 2017). *Porphyromonas Gingivalis* lipopolysaccharide (LPS) has been reported to contribute to the establishment of sustained periodontal inflammation and has been associated with some inflammatory processes with various systemic diseases (Jia et al., 2019; Xu et al., 2020).

Previously, we have mentioned the role of IL-17 signaling in promoting sustained immune responses and unresolved inflammatory states. IL-17 cascading has been also associated with the creation of environmental conditions leading to microbial dysbiosis. It has been recently analyzed how IL-17 may induce a microenvironmental shift toward highly pathologic bacterial settings, able to enhance periodontal inflammation (Bartold and Van Dyke, 2019). In addition, it has been discussed that IL-23-dependent IL-17 signaling promotes bacterial overgrowth, contributing for instance, to the establishment of the leukocyte adhesion deficiency periodontal phenotype. In contrast, IL-17 cascade inhibition stops bacterial overgrowth in periodontitis (Hajishengallis, 2015; Bunte and Beikler, 2019).

### 1.2. Periodontal Inflammation and Its Role in Cellular Physiology

**Figure 2:**
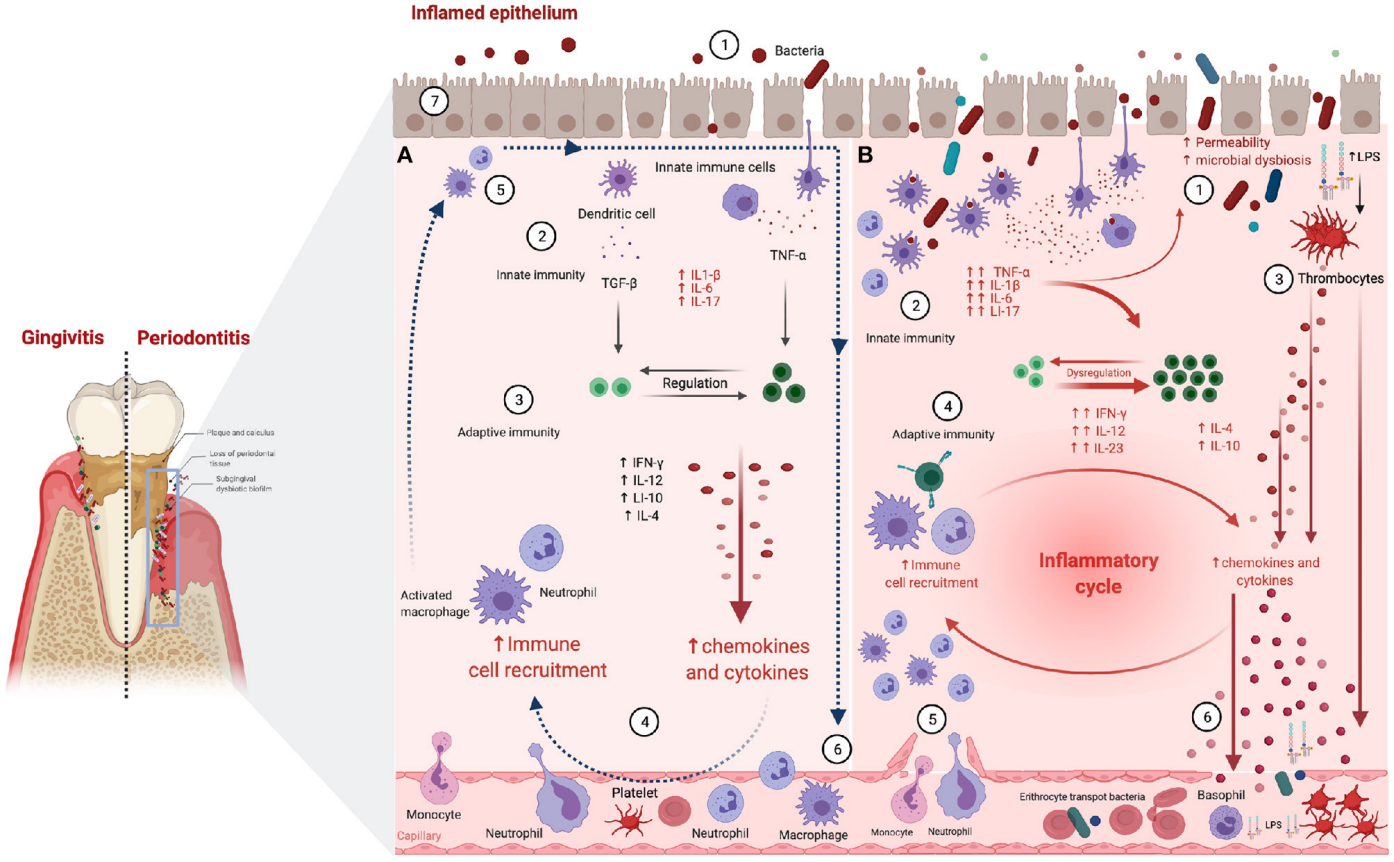
Periodontitis-associated immune and inflammatory processes. **(A)** Resolved inflammation and infection clearance scenario: ① Epithelial permeability and periodontopathogens entry. ② Innate immune cells detect LPS leading to pro-inflammatory cytokine production. ③ T cells are activated enhancing innate response via macrophage and neutrophil activation. ④ Macrophages and neutrophils release pro-inflammatory cytokines increasing vascular permeability. ⑤ Neutrophils clear periodontopathogens via infiltrating monocytes/macrophages, releasing tissue healing factors. ⑥ Neutrophils and macrophages exit the inflammation site and move back into the bloodstream. Pro-resolution mediators restrain immune cell influx, reversing vascular permeability, and coordinating the clearance of inflammatory debris. ⑦ The progression of acute into chronic inflammation is limited. **(B)** Unresolved periodontal inflammation and infection remaining scenario: ① Greater epithelial permeability and massive entry of periodontopathogens. ② Innate immune cells detect LPS, leading to pro-inflammatory cytokine production and phagocyte activation. ③ LPS activates thrombocytes, enhancing antimicrobial peptides and cytokine production. ④ Adaptive immune cells activate macrophages and neutrophils releasing pro-inflammatory cytokines. ⑤ Vascular permeability increases and leukocyte influx persist in inflamed periodontal tissues, due to failure of infection clearance, followed by the establishment of chronic inflammatory lesions. ⑥ Pro-inflammatory mediators and antimicrobial peptides are dumped into the circulation and may trigger inflammation in remote sites [Figure generated with BioRender (Biorender.com)].

Tissular architecture and cell physiology are also involved in the onset and development of PD and CP. In particular, the gingival epithelium works as a mechanical barrier to prevent and diminish bacterial invasion (Figure 2A ①; Fujita et al., 2018). This epithelium is also relevant since it is the playground in which the innate immune response to infectious inflammation in periodontal tissue starts (Graves, 2008). Microbial stress impacts the gingival epithelial barrier by enhancing the decay of the junctional complex, thus inducing neutrophil migration via chemokine activity. Neutrophils also secret proteases disrupting the epithelial barrier of the junctional epithelium (Figure 2A ② → ③; Hajishengallis, 2020).

Neutrophil associated chemokine and protease activity leads to cell-cell interaction disorganization enabling tissular inflammation characteristic of the initial phases of PD (Figure 2A ②; Balta et al., 2017). It has been argued that the molecular cascades leading to cell-cell disruption start at multi-protein cell junction (MPCJ) complexes. These complexes are symmetrical structures filling the gaps between cells and are known to be essential for the maintenance of the physical and functional integrity of tissues (Silva et al., 2019). As in other organismal locations, oral epithelial cells are generally interconnected by tight junctions, adherens junctions, desmosomes, and gap junctions all related to MPCJ complexes (Fujita et al., 2018).

Tight junctions in particular, are fundamental for the compact sealing of cellular sheets. These cell layers control the paracellular ion flux and participate in the regulation of tissue homeostasis (Belibasakis et al., 2015). Claudins are tight junction-associated proteins involved in the balance of the epithelial barrier. Claudin-1 is a common constituent of healthy junctional epithelium, becoming a key player of the epithelial barrier function. E-cadherin is an essential molecule in the formation of adherence junctions, helps to protect the tissues against bacterial invasion and low levels of this protein are often linked to inflamed gingival tissue (Fujita et al., 2010). Claudin and cadherin cascades become relevant to PD and CP since major periodontal pathogens are known to have the ability to decrease E-cadherin expression thus increasing oral epithelial permeability (Fujita et al., 2018; Figure 2B ①).

Structural remodeling of tissues also occurs at the level of the gap junctions via connexin deregulation (Figure 2A ⑥ → ⑦). Normal gap junctional intercellular communication (GJIC) is key to cellular coordination in tissue homeostasis (Figure 2A ⑦; Kato et al., 2013). Thus, abnormal GJIC responses of gingival epithelial cells (Figure 2B ①) secondary to the presence of periodontal pathogens (Figure 2B ② → ④) have been associated with the onset of periodontal disease (Fujita et al., 2018; Liu et al., 2020).

The gingival epithelium becomes also affected by the anomalous presence of apoptotic processes (Listyarifah et al., 2017; Carvalho-Filho et al., 2019). Apoptosis drives the destruction of the epithelial barrier function, thus enabling the onset and progression of PD (Li et al., 2019b; Taskan et al., 2019). Both pro and anti-inflammatory signals activated by microbial pathogens may induce apoptosis followed by an alveolar bone loss. The blockade of transforming growth factor (TGF)β/smad2 signaling has been regarded as a potential way to alleviate periodontal disease by allowing the recovery of the integrity of the gingival epithelial barrier (Fujita et al., 2018).

Epithelium remodeling also occurs secondary to leukocyte migration (Figure 2B ⑤). IL-8 levels in periodontal tissue and gingival crevicular fluid have been associated with the severity of PD and the development of CP. It has been argued that this is due to the role of IL-8 in the migration of polymorphonuclear leukocytes (Hajishengallis and Moutsopoulos, 2016). In this regard, PD development may be triggered by the progression of inflammatory cell infiltration in periodontal tissues (Figure 2B ⑥; Fujita et al., 2018). This phenomenon can be further enhanced, progressing to less-controlled cell death mechanisms such as pyroptosis (Cheng et al., 2018).

Remarkably, polymorphonuclear neutrophils (PMNs) as primordial innate immune defenders recruited constitutively to healthy mucosal sites partake in the initiation of immune cascades (Figure 2 ② → ④, both panels) (Daiuto et al., 2010). Then, locally-produced cytokines enter the bloodstream enhancing PMN production in the bone marrow, and increasing the mobility of circulating PMNs. Activated PMNs thus acquire tissue migration capabilities and travel across the endothelial barriers both in healthy mucosal tissues and at sites of inflammation (Fine et al., 2020).

Constitutive recruitment of PMNs to the healthy oral cavity is a normal physiological response, useful to hold the commensal biofilm at the interface between the gingival crevice and the tooth. In PD, however, PMNs reproduce at much higher rates. PMN increase has been associated with a *hyperactive phenotype* in which increased phagocytosis and degranulation, and higher production of reactive oxygen species (ROS) and neutrophil extracellular traps may coexist (Silva et al., 2019; Fine et al., 2020). Enhanced PMN reaction to oral pathogens often includes upregulation of pro-inflammatory cytokines both in local tissues as well as in systemic circulation. However, inordinate recruitment and dysregulated function of PMNs have been associated with a broad class of chronic inflammatory diseases (Fine et al., 2020). Inflammation of the periodontal tissue, therefore, allows for an exacerbated innate immune response. Among the main drivers we can include peripheral PMNs which may be activated synergistically by simultaneous and remote inflammatory stimuli, thus contributing to the interaction between PD and other inflammatory conditions. We may argue then that PD effects may extend well beyond oral health, while at the same time revealing novel approaches to treat systemic inflammatory diseases associated with PD (Fine et al., 2020). Related to this is the observation that inflammatory cytokines such as TNF-α, IL-1, and IL-6, are often increased in patients with CP, either alone or in combination with other chronic diseases (Cardoso et al., 2018).

### 1.3. Periodontal Inflammation and Immune Signaling

As mentioned in the previous sections, an intermediate mechanism that lies between bacterial stimulation and tissue destruction is the emergence of a cytokine network, which stimulates inflammatory events. Deregulated immune responses characterized by inappropriate secretion of some pro-inflammatory and anti-inflammatory cytokines (Figure 2B ② → ④), may lead to periodontal tissue decay (Kim et al., 2017; Kinane et al., 2017). Furthermore, these locally produced cytokines can diffuse, reaching systemic circulation, thus perpetuating a generalized inflammatory state (see Figure 2B ⑥) (Seymour et al., 2007; Konkel et al., 2019; Cecoro et al., 2020).

These cytokines include chemokines, innate immune cytokines, and acquired immune cytokines (Graves, 2008). Many of them are produced by a broad range of cells—or even by more than one type of cell—. Furthermore, each cytokine may have a completely different role in the cascade of events (Borish and Steinke, 2003; Garlet, 2010). Some cytokines have both pro- and anti-inflammatory functions; the final effect may depend on the cellular source, target, their relationship with other cytokines or in the specific phase of the immune response they participate (Dessaune et al., 2018). In general, pro-inflammatory mediators are related to tissue destruction, while anti-inflammatory mediators often limit periodontal disease progression (Garlet, 2010). An overview of cytokines related to periodontitis is presented in Tables 1, 2.

**Table 1:** Brief overview of pro-inflammatory cytokines involved in periodontal disease.

**Cytokine**	**Pro-inflammatory action**
IL-1	Is involved in both innate and adaptive immune responses, activates T lymphocytes by enhancing the
	production of IL-2 **[A]**, augments B-cell proliferation, increases immunoglobulin synthesis, stimulates
	endothelial cell adherence of leukocytes, induces periodontal bone loss stimulated by pathogens **[B]**.
IL-1α	Assists IL-23 and IL-6 in the activation of Th17 and the expression of IL-17 **[A]**.
IL-1β	Is induced upon host-microbiota interaction and leads the activation of both Th1 and Th2 cells; is involved
	in the inflammatory cell migration and stimulation of bone resorption **[A,C]**.
IL-2	Is implicated in the Treg cells and NK cell activation and B and T cell promotion and differentiation **[A,B]**.
IL-5	Promotes accumulation of eosinophils through its ability to upregulate responses to chemokines, enhances
	cytotoxicity, prolongs eosinophil survival at inflammation sites by blocking apoptosis, also induces maturation
	of cytotoxic T lymphocytes and basophilic differentiation **[A,B]**.
IL-6	Participates in the Th17 cell-induced differentiation of CD4+ T cells, associated with inflammatory cell
	migration and **[B,D]**, is implicated in bone homeostasis through upregulation expression of the receptor
	activator RANKL in osteoblasts, leading to osteoclast differentiation and bone resorption **[C,D]**.
IL-7	Is implicated in the development of B and T lymphocytes, stimulates the proliferation and differentiation
	of cytotoxic T cells and NK cells, stimulates the tumoricidal activity of monocytes and macrophages **[B]**.
IL-9	Supports the growth of antigen-specific T lymphocytes and regulates some hematopoietic cells **[B]**.
IL-12	Promotes IFN-γ production in T cells and NK for bacterial clearance, activates and induces proliferation,
	cytotoxicity, as well as cytokine production of NK cells; IL-12 mediates the clearance of local bacteria,
	inhibits bone resorption displaying, also protective effects in the pathogenesis of PD **[B,D]**.
IL-17	Recruits and activates cells of the innate immune response, upregulates antimicrobial factor expression
	leading to dysbiotic microbiome promotion **[D,E]**, exerts protective effects on the local mucosal barrier **[A]**.
	IL-17 is also associated with chronic inflammatory tissue destruction and alveolar bone loss **[B,C]**.
IL-18	Is an IFN-γ-inducing factor, and an activator of Th1 and NK cells, stimulating the expression of MMPs **[C,D]**.
	IL-18 overexpression is related to inflammatory bone loss after *P. gingivalis* infection, also drives the
	polarization and activation of antigen-specific lymphocytes and myeloid cells under microbial challenge **[D]**.
IL-23	Is a member of the IL-12 family, secreted by myeloid antigen presenting cells exposed to *P. gingivalis* and
	periodontal ligament fibroblasts stimulated by IL-1β **[B,C]**. Promotes the pathogenicity of Th17 cells and
	the suppression of anti-inflammatory IL-10 **[B]**.
IL-33	Is involved in the modulation of Th2 cells, related to periodontal bone loss **[B,C,D]**, drives the differentiation,
	polarization and activation of antigen-specific lymphocytes and myeloid cells under microbial challenge **[D]**.
IFN-γ	Stimulates antigen presentation and cytokine production by monocytes, stimulates the accumulation of
	macrophages **[B,D,E]**, killing intracellular pathogens and clearing infections **[F]** by NK cells and neutrophils.
	IFN-γ is also related to periodontal tissue destruction **[D]**.
TNF	Participates in bone metabolism, exacerbating bone resorption and damaging the oral mucosal barrier **[B,C]**.
	TNF-α plays a central role in the phagocyte cell activation and endotoxic shock **[F]**, loss of connective tissue
	attachment and up-regulates the production of classic pro-inflammatory innate immunity cytokines **[E]**.
	Along with IL-1β is associated with inflammatory cell migration and osteoclastogenesis processes **[D]**.

*IL, interleukins; Th, T helper; Treg, regulatory T cells; NK, natural killer; RANKL, receptor activator of nuclear factor-kB ligand; IFN-γ, interferon-γ; MMPs, matrix metalloproteinases; TNF, tumor necrosis factor; Reference sources: **[A]**: Pan et al. (2019), **[B]**: Borish and Steinke (2003), **[C]**: Ramadan et al. (2020), **[D]**: Garlet (2010), **[E]**: Graves (2008). **[F]** Turner et al. (2014)*.

**Table 2:** Brief overview of anti-inflammatory cytokines involved in periodontal disease.

**Citokine**	**Anti-inflammatory action**
IL-2	Is implicated in T-cell growth factor, in the effect on Treg cells as well as in the promotion of T lymphocytes,
	is associated with the activation of NK cells, B cells, cytotoxic T cells, and macrophages, IL-2 also acts as an
	inhibitory factor in periodontal disease development **[A,B]**.
IL-4	Stimulates the humoral immune response—via B-cells—, downregulates the production of IL-1, IL-6, and
	TNF-α **[A,B]**, induces the production of cytokines with similar or complementary suppressive properties
	such as IL-10, also inhibits the production of MMPs and RANKL, and inhibits Th1 and bone resorption **[C]**.
IL-10	Is related to the suppression of innate immunity cytokines, interferes with IFN-γ, and IL-17 production
	by T-cells **[A,B,C]**, presents a direct protective role in tissue destruction (suppression osteoclastogenesis),
	modulating MMPs and RANK systems **[C]**.
IL-11	Induces lymphoid cell differentiation and downregulates pro-inflammatory cytokines **[A,B]**, is a stimulatory
	factor for hematopoietic precursor cells, such as erythrocytes, platelets and mast cells, and for connective
	tissue cells, such as fibroblasts **[A]**.
IL-13	Shares some activities with IL-4 on mononuclear phagocytic cells, endothelial cells, epithelial cells,
	and B cells **[A]**.
IL-17	Participates in signaling downstream of IL-1 and TNF-α, is related to periodontal barrier integrity and control
	of the oral microbiota. Plays a double role in both local tissue homeostasis and the pathogenesis of PD **[B]**.
IL-27	Induces the expression of IL-10 in Th1 and inhibits the immunopathology of Th17 response **[A,B]**.
IL-37	Secreted from Treg cells that recently have been reported to suppress the function of NK cells **[A]**.
IFN-γ	It is the most important cytokine responsible for cell mediated immunity **[A]**, also inhibits osteoclast formation
	and helps to control periodontal infection **[C]**.
TGF-β	It is a key element for immune regulation **[B]**; inhibits the action of B lymphocytes and T-helper cells, as well as
	the cytotoxicity of phagocytes, lymphocytes and NK cells **[A]**; inhibits the production of IL-1β, TNF-α, and
	IL-6 **[C]**; down-regulates the transcription of MMPs contributes to tissue repair, stimulating collagen synthesis,
	neovascularization, and fibroblast proliferation **[D]**.

*IL, interleukins; Treg, regulatory T cells; NK, natural killer; TNF, tumor necrosis factor; MMPs, matrix metalloproteinases; RANKL, receptor activator of nuclear factor-kB ligand; IFN-γ, interferon-γ; TGF-β, transforming growth factor-β. References **[A]**: Borish and Steinke (2003); **[B]**: Pan et al. (2019), **[C]**: Garlet (2010); **[D]:** Dessaune et al. (2018)*.

Some cytokines are closely related to certain groups of T lymphocytes (naive CD4+ T cells) which under inflammatory cytokine's stimuli are differentiated into Th1, Th2, Th17, Tfh (follicular helper T cells), and Treg cells. Of these, Th1 (IL-12, IFN-γ) and Treg (IL-2 and TGF-β, IL-10 family), have been associated with pleiotropic and anti-inflammatory effects in periodontitis; Th17 (IL-17 and IL-23) and Th2 (IL-4, IL-5, IL-13) also have been associated with pleiotropic effects. Th2 cells have been related to destructive B cell response in chronic periodontitis (Pan et al., 2019). Th17 cells have been shown to be potent inducers of tissue inflammation and have been associated with the pathogenesis of many immune-mediated inflammatory diseases (psoriasis, rheumatoid arthritis, multiple sclerosis, inflammatory bowel diseases, and asthma) (Miossec and Kolls, 2012; Noack and Miossec, 2014; Chen and Kolls, 2017).

Due to extensive signaling crosstalk, innate immune activity pathways may alter the way adaptive immunity works, these two major routes of immunity are able to act in a highly coordinated manner in order to establish, maintain and, when needed, restore tissue homeostasis. In view of this, any process inducing dysregulation of this coordinated action may result in chronic inflammation (Bunte and Beikler, 2019). This is the case of sustained infectious states such as the ones present in CP. In this regard, cells of the adaptive immune system such as Th1 and Th2 are known to play major roles in the pathogenesis of immune-mediated inflammatory diseases. In addition, there has been a recent interest in the effects of Th17 cells, as their signals via IL-17 and IL-23, are able to induce large changes in the innate immunity regulatory pathways. Given that innate immunity triggers initial acute inflammatory responses to tissue injury, trauma, or pathogens, its action is able to promote phagocytosis, and activate the complement and adaptive immune systems with long run consequences (Hajishengallis, 2015; Fine et al., 2020). In contrast with the rather nonspecific innate immunity responses, once the adaptive immune system is activated, it results in a cascade of antigen specific host responses mediated by both, B and T cells (Wang et al., 2016).

Tracing back the origins of CP, we may start by considering the dysbiosis of oral microbiota and pro-inflammatory events involving both immune cells and molecular mediators of innate and adaptive immunity responses (Figure 1; Pietiäinen et al., 2019). These events lead to chronic inflammation of periodontal tissues driven by interactions within hybrid cytokine networks: pro-inflammatory cytokines, including IL-1α, IL-1β, TNF-α, IL-6, and IL-17 contribute to acute and chronic inflammation and tissue injury, while a second group formed by cytokines such as IL-10 leads to antagonistic effects (Table 2; Cardoso et al., 2018). Further unbalances in the immune cascades are able to induce immune-mediated cell differentiation. Under sustained infection, chronic inflammatory responses may induce differentiation of CD4+ cells unto the Th17, a lineage characterized by high levels of IL-17 production. It is worth recalling that Th1 and Th2 cells are not able to signal for IL-17 release. There are still many unknowns in the way these signaling and differentiation cascades proceed, however, it is known that Th17 cell production is mediated by a complex cytokine and transcription factor regulatory network comprising several of the known inflammatory pathways associated with autoimmune conditions (Chen and Kolls, 2017). IL-17 release is of course of great relevance to understand systemic inflammation, since a wide variety of cell types such as osteoblasts, endothelial cells, epithelial cells, fibroblast-like synoviocytes, chondrocytes, fibroblasts, keratinocytes, and macrophages are able to express the IL-17 receptor (Noack and Miossec, 2014; Bostanci et al., 2019).

Cell biology studies have shown that IL-17 producing cells—including Th17 and NK cells, among others—grow and accumulate more often on mucosal surfaces such as the oral cavity, the gastrointestinal tract, as well as the lung, vagina, and skin epithelia (Bunte and Beikler, 2019). This fact is relevant in view that IL-17 signaling is able to regulate protective immunity against extracellular pathogens by sustaining barrier integrity, promoting the release of antimicrobial factors, as well as activating granulopoiesis (Duka et al., 2019). Along with IL-17, the Th17-produced IL-22 is able to enhance IL-17 protective functions by improving antimicrobial peptides synthesis and neutrophil uptake (Hajishengallis, 2020). Hence, any processes, resulting in inhibition of IL-17 production or signaling may enhance susceptibility to bacterial and fungal infections (Bunte and Beikler, 2019). A number of actual mechanistic explanations have been advanced, for instance, Bostanci and collaborators analyzed—via an experimental model of periodontitis—the role of TREM-1 (Triggering receptor expressed on myeloid cells 1, a protein that amplifies inflammatory processes) as an over-expressed gene whose blockade inhibits IL-17A and RANKL transcription thus diminishing bone loss (Bostanci et al., 2019).

In connection with the role of Th17 cells and immune responses at the tissular scale, it has been argued that since Th17 cells are commonly anchored in the gingival tissues, they may play a role in protecting the oral barrier. The potential mechanisms are not yet understood, however, in an animal model, IL-17R- mice have been shown to be more prone to *P. gingivalis*-induced bone loss (Yoshihara-Hirata et al., 2018). This suggests that IL-17 may play a protective role at the tissular level, in particular in bone remodeling and homeostasis. Interestingly, a number of clinical studies in humans have shown that excessive production of IL-17 is associated with periodontitis. Similar protective roles have been shown in the case of FICZ (tryptophan photooxidation product 6-formylindolo[3,2-b]carbazole, a physiological ligand for the mammalian aryl hydrocarbon receptor to which it binds inducing expression of cytochrome P450) and molecules in the aryl hydrocarbon receptor axis in relation to alveolar bone loss and inflammation in experimental (murine) periodontitis (Huang et al., 2019).

This apparent paradox is indeed unsurprising once we consider the known functions of IL-17 (Tables 1, 2). On the one hand, IL-17 is actually a weak inducer of inflammation, that only acquires enhanced inflammatory potential via synergistic effects with other cytokines and via neutrophil activation (Pan et al., 2019). Moderated effects in this regard, bring immune protection and inflammatory resolution. On the other hand, extensive upregulation of IL-17 may lead to excessive activation and mobilization of neutrophils as well as increased production of chemokines signaling for neutrophil diapedesis (Ramadan et al., 2020), thus promoting tissue damage. Furthermore IL-17 in combination with IL-1 triggers CCL20 production from human gingival fibroblasts stimulating recruitment of Th17 cells; leading to a feed-forward loop of IL-17 production (Bunte and Beikler, 2019; Zekeridou et al., 2019). Other chemokine-associated pathways are also relevant to CP. Kavrikova et al. (2019) have extensively discussed the role that the chemokine receptor CXCR2 and its molecular variants play in sustained infectious states and unresolved immune responses in the context of CP. It has also been reported that resolvin E1 is able to reverse the effects of periodontitis and dysbiosis in experimental models (Lee et al., 2016; Balta et al., 2017).

### 1.4. The Genetics of Periodontal Inflammation

Genetic factors associated with the susceptibility, severity and the evolution of periodontitis have pointed out to a number of variants in genes of interest (Haworth et al., 2021; Shaddox et al., 2021). Relevant associations have been found, for instance, in genetic polymorphisms in genes such as IL1B, IL1RN, FcγRIIIb, VDR, and TLR4 in connection to susceptibility to aggressive periodontitis; and in IL1B, IL1RN, IL6, IL10, VDR, CD14, TLR4, and MMP-1 related to chronic periodontitis (Laine et al., 2012). Heritability of susceptibility to periodontal disease has also been discussed. A study in monozygotic and dizygotic twin pairs has estimated a heritability as high as 50% after adjusting for behavioral and environmental features. Interestingly, no evidence of heritability has been found in gingivitis (considered a precursor condition for periodontitis, see Figure 1B) after adjusting for these environmental covariates (Michalowicz et al., 2000).

Genome wide association studies (GWAS) have also provided with important clues on the genetics of periodontal disease, including aspects related to heritability dependencies, showing that for related conditions such as dental caries heritability is enriched in conserved genomic regions (Shungin et al., 2019). Most of the loci associated with PD in GWAS however, correspond to non-coding genomic regions and are hence, thought to be involved in modulating gene expression via regulatory interactions. As in most GWAS approaches, identifying the causal variants behind these associations remains challenging; this in turn makes difficult to elucidate the molecular mechanisms, useful for diagnostics and therapeutics and ultimately linked to a deeper understanding of how these variants contribute to phenotype establishment and clinical traits (Schaefer, 2018). In the case of genomic regions with known genetic factors in PD, a systematic review and meta-analysis with more than 70,000 participants describes genetic variants in genes such as IL-1A, IL-1B, IL-6, IL-10, MMP-3, and MMP-9 were significantly associated with the risk of developing periodontitis (da Silva et al., 2017).

Some additional insight has been found in studies in which PD is analyzed in combination with other conditions, such as cardiovascular diseases. It has been hypothesized that, since PD and coronary artery disease (CAD) are both characterized by overaggressive inflammatory responses to stimuli, they may share some genetic background. By analyzing joint conditions, association studies have found that genes like ANRIL/CDKN2B-AS1, PLG, CAMTA1/VAMP3 are involved in the pathogenesis of PD and CAD, a fact that suggests functional features and helps to account for some of the missing heritability of PD (Aarabi et al., 2017).

Combined genetic and functional studies point to immunogenetic blueprints in which immune fitness is disturbed, which has led to the proposal of a signature of more than 65 genes, involving inflammatory features and (again) association with cardiovascular diseases. In particular, four genetic loci have been revealed: CDKN2B-AS1(ANRIL), a conserved noncoding element within CAMTA1 upstream of VAMP3, PLG, and a haplotype block at the VAMP8 locus (Loos and Van Dyke, 2020). GWAS of aggressive PD have in turn revealed the role of variants in other genes such as GLT6D1, DEFA1A3, and SIGLEC5, also clearly associated with host immune responses (Masumoto et al., 2019).

Genes associated with bone morphogenetic and developmental processes have also been associated with PD. In particular, in the case of persistent apical periodontitis, Single nucleotide polymorphisms (SNPs) in the BMP2, BMP4, SMAD6, and RUNX2 genes were significantly associated and suggested epistatic interactions, in particular SNP-SNP interactions leading to important increased risk (odds ratios up to 4.36 in the high risk genotypes) (Küchler et al., 2021).

Aside from localized polymorphisms such as SNPs, larger genetic variants have been linked to PD. For instance, long runs of homozygosity (LROH) were associated with different stages of PD; in particular LROH spanning as long as 33 genes are significantly associated with an increased severity of periodontitis (Mezzavilla et al., 2021). Some of these genes have been previously related to granulocyte and platelet developmental processes.

The relation between microbial infections and hosts is complex, even more so in the case of chronic infections such as the ones responsible for PD and especially CP. A myriad of complex molecular mechanisms are often involved in triggering and maintaining infection-associated inflammation (Ari et al., 2016). For instance, Leukocyte adhesion deficiency-1 (LAD1) is a genetic immunodeficiency caused by a mutation leading to defective neutrophil adhesion and tissue transmigration. LAD1 produces recurrent skin infections, as well as oral ulcers, severe periodontal inflammation, and bone loss (Shaddox et al., 2017). Severe PD in LAD1 patients had been traditionally attributed to deficient neutrophil surveillance in gingival and periodontal tissues. However, recent evidence that enhanced inflammatory response mediated by IL-17, either due to genetic origins—such as eQTL enhancing—or signaling unbalance of infectious or immune origins, is also closely involved (Bunte and Beikler, 2019). Genetic variants in the chemokine receptor CXCR2 have been associated, for instance, with susceptibility to prolonged periodontal bacteremia leading to chronic periodontitis (Kavrikova et al., 2019).

Methods of genetic analysis have also revealed intriguing aspects of periodontitis and its comorbidities. In a landmark study, Czesnikiewicz-Guzik et al. (2019) were able to found a causal association between periodontitis and hypertension by resorting to both, Mendelian randomization and a randomized controlled trial of non-surgical periodontal therapy. There have been other efforts to analyze the relationship between PD and hypertension (Pietropaoli et al., 2020). Two large surveys were analyzed in this regard by Leira et al. (2020b). Unsurprisingly, the authors found that systemic inflammatory states were at the center connecting the two conditions.

### 1.5. The Epigenetics of Periodontal Inflammation

These mechanisms also include epigenetic pathways driven by upstream regulations, as well as other processes that may be traced back to its genetic and epigenetic origins (Ari et al., 2016). Epigenetic modifications, in particular, include chemical modifications of DNA and proteins, changes affecting chromatin remodeling which may result in the activation or inactivation of transcription cascades thus deregulating gene expression (Shaddox et al., 2017). The crucial role of DNA and histone modifications, two of the major epigenetic regulatory processes, have been documented to occur in the development of periodontitis (Diomede et al., 2017). Host-pathogen interactions are often mediated by molecules such as LPS. LPS-associated responses trigger a cascade of chronic inflammatory events and are able to modulate host responses at the cellular and tissular levels (Diomede et al., 2017). These responses produce alterations in DNA methylation patterns, thus modifying the expression programs of immune-related genes leading to inflammatory disease progression (Palioto et al., 2019). DNA methylation and histone acetylation are the major epigenetic modifications induced by diseases and environmental factors. DNA (cytokine-5) methyltransferase 1 (DNMT1) and histone deacetylases (HDACs) are the key regulators of DNA methylation and histone acetylation, respectively, and are known to be shaped by the action of pathogens such as *P. gingivalis* (Diomede et al., 2017).

It has been reported that histone acetylation promotes the transcription of inflammatory genes such as p300/CBP histone acetyltransferase, NF-κB and other pro-inflammatory cytokines in PD (Ramadan et al., 2020). The extent to which this happens during PD progression, however, remains unclear. NF-κB signaling is thought to be involved via the sustained regulation of histone modifications accelerating disease progression. NF-κB plays a central role by activating innate immunity pathways driving osteoclast differentiation and inducing bone resorption (Fine et al., 2020). DNA methylation is regulated here by two different types of DNA methyltransferases (DNMTs): *de novo* methyltransferases DNMT3a and DNMT3b, active during early development; as well as *maintenance* methyltransferase (DNMT1), which keeps methylated and unmethylated CpG sites in the cells (Diomede et al., 2017).

Epigenetic regulation of inflammation has been studied in the context of periodontitis by Jurdziński et al. (2020). These authors argue that a deeper understanding of these epigenetic regulatory mechanisms will provide clues about functions at the cellular level with potential therapeutic impact. Other functional epigenomic regulation phenomena in PD are mediated by non-coding RNAs, in this regard, Jin et al. (2020) have identified a group of novel PD-associated lncRNAs by performing a weighted gene co-expression network analysis.

## 2. Medical Aspects of Periodontal Inflammation

**Figure 3:**
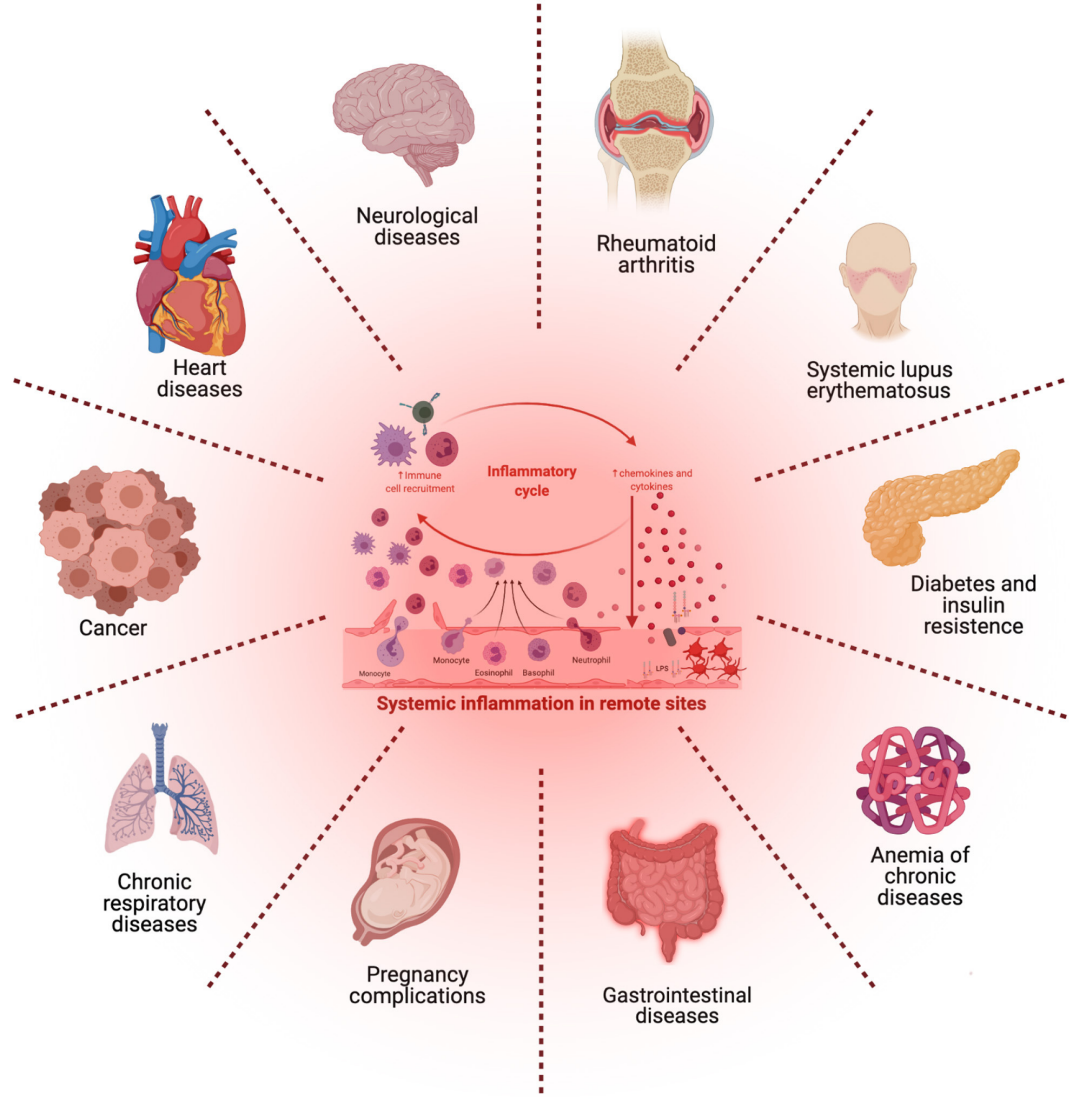
Microbial imbalance, irregular immune response, and chronic periodontitis may exert systemic or distant effects and could lead to the activation of other disorders that share common inflammatory pathways. [Figure generated with BioRender (Biorender.com)].

PD and CP are implicated in a multitude of pathogenic processes (see Figure 3), for this reason their prevention is essential for public health (Fujita et al., 2018). As previously discussed, it has been argued whether specific periodontal pathogens are able to stimulate and trigger for the development of systemic inflammatory disease, or if it is actually systemic disease the one leading to microbiome dysbiosis causing an abnormal abundance of periodontal pathogens. If, as is often the case, these same *oral* pathogens are able to induce non-oral diseases, they may become targets for therapeutic intervention, perhaps via drug repurposing schemes. In any case, the presence of periodontal pathogens may at least be used as a diagnostic marker to predict susceptibility to non-oral disease (Bui et al., 2019).

### 2.1. Periodontal Inflammation: Its Relation With Chronic Diseases

In recent years, several research and clinical studies have reported associations between periodontitis and a number of systemic inflammatory conditions, including arthritis, type 2 diabetes mellitus, and atherosclerosis (de Molon et al., 2019). The infectious, molecular and physiological origins of these associations have been discussed in the previous sections. Now, we will proceed to discuss their consequences in the onset and development of a broad range of diseases, including cardiovascular disease, gastrointestinal and colorectal cancer, diabetes and insulin resistance, Alzheimer's disease, as well as respiratory tract infection and adverse pregnancy outcomes among many others (Bui et al., 2019; Khumaedi et al., 2019).

The relationship between CP and other systemic chronic inflammatory diseases will gain relevance since a number of therapeutic interventions such as cytokine-based treatment strategies with the potential to improve both CP and systemic health exists (Cardoso et al., 2018). Immune markers linking CP and diabetes have also been reported in relation to glycation dynamics and TNF-α, which have been argued to constitute reliable biomarkers of inflammation in gingival crevicular fluid and serum (Singhal et al., 2016). Glycemic status has been associated with periodontitis via mechanisms of systemic inflammation (Torrungruang et al., 2018). Bone marrow stem cells co-cultured with macrophages obtained from diabetic periodontitis patients have shown an interplay between local inflammation and innate immune responses (Wang et al., 2016) further highlighting the relationship between these two conditions. On the other hand, there is indeed evidence which supports the fact that periodontal treatment alleviates systemic inflammation in type 2 diabetes (Preshaw et al., 2020).

A recent experimental (mouse) model, for instance, showed how periodontitis is able to induce systemic inflammation leading to atherosclerotic exacerbation via a mechanism that drives endothelial—mesenchymal transitions (Suh et al., 2019; Schenkein et al., 2020). Similar mechanisms have also been discussed in the context of the clinical care of human patients with lacunar infarct (Leira et al., 2019b).

#### 2.1.1. Cancer

Another set of complex diseases for which increased risks have been associated with CP is cancer (Galvão-Moreira and da Cruz, 2016). It is known that compared to individuals with no PD, fatal cancer is positively associated with periodontitis, with lung cancer being strongly associated—though the actual relationship is still debated—(Hujoel et al., 2003), while oral and esophageal carcinomas resulted more consistently correlated (Fitzpatrick and Katz, 2010; Shin et al., 2019).

Particularly common instances are oral cancers of epithelial origin and squamous cell carcinoma (Chung et al., 2019; Irani et al., 2020). Indeed, it has been suggested that the risk of oral squamous cell carcinoma (OSCC) could be modulated by reducing periodontitis. There is also an increasing interest in the link between PD and overall cancer risk, in particular for digestive tract tumors, pancreatic, prostate, breast, cervix, oropharingeal, and lung cancers, as well as Non-Hodgkin lymphoma (Dizdar et al., 2017; Corbella et al., 2018; Michaud et al., 2018). For breast cancer, a meta-analysis of close to 2-million subjects found PD significantly associated with the development of breast cancer, though in patients with a history of PD with periodontal treatment risk ceased to be statistically significant (Shao et al., 2018). To further stress the role of oral hygiene, increased risk for several cancers has been associated not only with PD, but also with periodontitis leading to tooth loss (Maisonneuve et al., 2017; Michaud et al., 2017).

As in other diseases that we will discuss later, systemic inflammation is also thought to be serving a key biological role (Cardoso et al., 2018). It is a well-established fact that inflammation is able to profoundly affect all phases of cancer (Table 3). Inflammatory and immune mediation processes are well known hallmarks of cancer, starting from the onset at the single-cell level, all the way up to early tumor growth, progression, and dissemination of neoplasms. Another emerging concept, in this regard, is that cancer, much like other complex diseases, is actually the consequence of systemic, rather than local conditions. Systemic inflammation is in itself quite a complex phenomenon involving the interplay of functional relationships with the deregulation of energetic metabolism and immune signals, that together with genetic instability, predispose individuals to develop cancer and regulate the abnormal states that sustain neoplastic disease (Cardoso et al., 2018).

**Table 3:** Brief overview of chronic diseases related with periodontal disease.

**Disease**	**Pathophysiology**
Cancer	Abnormally high signaling levels of TNF-α, IL-1, and IL-6 in CP promote oncological diseases.
	Prolonged exposure of periodontopathogens (*Pg, Fn, Cr, Cd*) activates tumorigenesis-related
	pathways, increases aggressiveness of cancer cells and promotes tumor progression **[A-E]**.
Metabolic	TNF-α and IL-6 are related to insulin resistance. Insulin resistance may promote both higher
syndrome	cytokine concentrations and systemic oxidative stress **[F–H]**.
Diabetes	Chronic hyperglycemia decreases macrophage and neutrophil function **[I]**.
(BA)	PD is able to induce systemic inflammation leading to atherosclerotic exacerbation **[J,K]**.
	Acute bacterial infection (*Pg, Aa, Fn, Pc*) increases long-lasting insulin resistance **[B,L]**.
	IL-6 acts as a major procoagulant cytokine and may also activate hepatocytes, thus enhancing
	the production of acute phase reactants and other inflammatory mediators **[N]**.
Obesity	Metabolic endotoxemia of periodontopathogens may contribute to the development of obesity **[G]**.
	PD increases systemic LGI driving exacerbated gene expression of hepatic levels of TNF and IL-6 **[M]**.
	PD-associated systemic inflammation can inhibit the insulin receptor, promoting insulin resistance **[G]**.
Atherosclerosis	Inflammatory cytokines are secreted from adipocytes and macrophages in the adipose tissue **[A]**.
	TNF-α and IL-6 are associated with endothelial dysfunction leading to impaired vasodilation
	and alterations in the vascular structure **[M]**. Periodontopathogens (*Pg*) promotes atherosclerotic
	plaques induce platelet aggregation and vascular inflammation **[B,D,O,P]**.
CVD	Bacteremia (*Pg, Aa, Tf, Ec, Fn, Pi, Td,Cr*) and systemic inflammation associated with CP are
	important factors in the initiation of the endothelial lesion and can induce platelet aggregation **[A,B]**.
	Vascular inflammation is modulated by pro-inflammatory cytokines such as TNF-α, IL-1, and IL-6 **[Q]**.
Respiratory	Pathogens (*Pg, Aa, Ec, Fn, Pi, Cp, Ai, Cs, Sc*) aspirated into the lower respiratory tract promote a
diseases	pro-inflammatory microenvironment **[A,B]**. The virus enters in the circulation from periodontal pockets
	via gingival crevicular fluid or via periodontal capillaries **[R,S]**. PD co-infection with virus like
	SARS-CoV-2 can aggravate inflammatory response and cytokine storm **[T]**.
Rheumatoid	Dysbiotic microbiota of periodontitis translocates from the oral cavity to the synovium **[E]**.
arthritis	In the citrullination process, *P. gingivalis* leads to autoantibody (anti-citrullinated protein antibodies)
	formation and compromised immune tolerance of patients susceptible to rheumatoid arthritis **[U,V]**.
	Abnormal immune response and increased secretion of pro-inflammatory mediators
	(TNF, IL-1β, IL-6, IL-22, IL-23, and IL-17) results in synovium tissue destruction **[W,X]**.
Neurodegenerative	Periodontopathogens (*Pg, Aa, Fn, Pi, Td, Cp*) may enhance development of brain inflammation **[B,Y,Z]**,
(BA)	which appears to be one of the main drivers of neurodegeneration in Alzheimer's disease **[B,Aa]**.
	Some periodontopathogens may invade the brain by crossing the brain-blood barrier **[B,Aa]**.
Obstetric	Periodontopathogens (*Pg, Aa, Tf, Ec, Fn, Pi, Td, Pn*) and their products into the circulation and to
(BA)	the placenta may stimulate immune and inflammatory responses **[A,B, Ab]**
	leading to a high secretion of pro-inflammatory cytokine levels in the fetal tissues **[E,U]**.

*BA, Bidirectional association; Pg, Porphyromonas gingivalis; Fn, Fusobacterium nucleatum; Cr, Campylobacter rectus; Cd, Clostridium difficile; Aa, Actinobacillus actinomycetemcomitans; Tf, Tannerella forsythia; Ec, Eikenella corrodens; Pi, Prevotella intermedia; Td, Treponema denticola; Cr, Campylobacter rectus; Cp, Chlamydia pneumoniae; Pn, Prevotella nigrescens; Ai, Actinomyces israelii; Cs, Capnocytophaga spp.; Sc, Streptococcus constellatus. References: **[A]**: Cardoso et al. (2018); **[B]**: Bui et al. (2019), **[C]**: Fine et al. (2020), **[D]**: Gallimidi et al. (2015), **[E]**: Ha et al. (2015), **[F]**: Lamster and Pagan (2017), **[G]**: Jepsen et al. (2020), **[H]**: Minty et al. (2019), **[I]**: Torrungruang et al. (2018), **[J]**: Suh et al. (2019), **[K]**: Schenkein et al. (2020), **[L]**: Singhal et al. (2016), **[M]**: Cecoro et al. (2020), **[N]**: Baeza et al. (2020), **[O]**: Furutama et al. (2020), **[P]**: Ferguson et al. (2020) **[Q]**: Preshaw et al. (2020), **[R]**: Sukumar and Tadepalli (2021), **[S]**: Badran et al. (2020), **[T]**: Coke et al. (2021), **[U]**: Han and Wang (2013), **[V]**: González-Febles and Sanz (2021), **[W]**: Maldonado et al. (2020), **[X]**: Bunte and Beikler (2019), **[Y]**: Poole et al. (2013), **[Z]**: Poole et al. (2015), **[Aa]**: Stein et al. (2012), **[Ab]**: Bobetsis et al. (2020)*.

A meta-analysis including 3,183 subjects demonstrated that patients with PD present increased susceptibility to oral cancer. More recent studies have actually discovered a positive correlation between PD and pancreatic, head and neck, and lung cancers (Galvão-Moreira and da Cruz, 2016). Another study examined one million randomly selected insurance cases in Taiwan, and found that patients in one periodontitis cohort had an elevated risk of developing cancer as compared with the one of those in a comparable gingivitis cohort (Bui et al., 2019). Furthermore, the periodontal pathogen *P. gingivalis*—which has been strongly associated with the development of both, PD and CP—was found to be highly abundant in OSCC and esophageal squamous cell carcinoma (ESCC) patients (Bui et al., 2019). Further studies have also advanced clues in this direction. For instance, the potential role that periodontal pathogens, such as *P. gingivalis* and others may be playing in the onset and development of oral cancers, was inferred from an animal model for oral-specific chemical/microbial carcinogenesis. *Porphyromonas gingivalis* and *Fusobacterium nucleatum* were shown to stimulate tumorigenesis through a mechanism involving direct interaction with oral epithelial cells. This mechanism is mediated via the host innate immune system (Fine et al., 2020).

Periodontitis has also been associated with cancer mortality in a 10-year follow up study of a cohort with 68,273 adults. In particular, it has been suggested that low grade chronic inflammation linked to PD may influence carcinogenesis (Heikkilä et al., 2018). PD-associated cancer mortality may be indeed related to immune responses. In breast cancer, for instance, it has been argued that periodontal inflammation favors recruitment of distant metastatic breast cancer cells by increasing myeloid-derived suppressor cells in an experimental model (Cheng et al., 2020).

Regarding OSCC, *P. gingivalis* was able to promote invasion and metastasis of oral squamous cells by induction of MMP-9 expression. It was also demonstrated that long, repetitive exposure to *P. gingivalis* enhances the aggressiveness of oral cancer cells by inducing epithelial-mesenchymal transformations in the cells and that *F. nucleatum* triggers a pro-inflammatory microenvironment, thus enhancing tumor progression in colorectal adenoma-carcinoma (Bui et al., 2019). Since the elevated presence of *P. gingivalis* and *F. nucleatum* positively correlates with the onset of oral cancer, it has been proposed that they could be used as early-stage biomarkers for the oncological disease, or even more as targets for prevention of (some) oral cancers in humans (Bui et al., 2019).

#### 2.1.2. Metabolic Diseases

Aside from neoplastic conditions, PD and chronic inflammatory processes are known to be closely related comorbidities with metabolic diseases. For instance, there has been documented that CP and diabetes are associated conditions. This association is actually believed to have causal components and indeed it has been described as a *bidirectional* association: Diabetes is a known modifying factor for chronic CP. CP on the other hand, is a common complication of diabetes (Chi et al., 2010; Cardoso et al., 2018). Some mechanistic hints have already been disclosed: the physiological processes involved in the onset of disease have been related to chronic hyperglycemia unleashing cascades of decreased macrophage and neutrophil function, accumulation of advanced glycosylation, and inflammation (Torrungruang et al., 2018). Concomitant presence of periodontitis is on its part, known to impair glycemic control in diabetics, hence increasing the risk for diabetes complications and comorbidities. From the signaling perspective: Pro-inflammatory mediators, like TNF-α, IL-1, and IL-6 that are upregulated in both diseases, a fact that may point to common molecular origins (Table 3). Furthermore, interindividual variability in both diseases may be influenced by sharing genetic, epigenetic, and environmental factors (Cardoso et al., 2018).

Prolonged infection during periodontitis can drive exacerbated and dysregulated inflammatory responses. These, in turn, may contribute to poor metabolic control of blood sugar and increased insulin requirements (Purnamasari et al., 2019). It has been shown that individuals with acute bacterial and viral infection present severe and long-lasting insulin resistance (Singhal et al., 2016). This phenomenon was further confirmed in a study with 124 middle-aged men, concluding that high titers for enteroviruses and *Chlamydia pneumoniae* correlated strongly with insulin resistance, quite likely due to chronic LGI resulting from these infections. In the case of *P. gingivalis*, a decrease in gingival vascular function and increased insulin resistance was observed in a murine diabetes model (Bui et al., 2019).

The explicit mechanistic details of these associations are still missing, however, CP appears to increase the risk of diabetes via infectious and inflammatory responses. On the other hand, long term diabetes may lead to a number of inflammation-related conditions: poor wound healing, retinopathy, nephropathy, neuropathy, macrovascular disease, and periodontitis. Diabetics actually show a threefold risk increase for periodontitis, as compared with non-diabetic subjects (Bui et al., 2019). Periodontitis, diabetes and LGI were explored in-depth and discussed in the review paper by Cecoro et al. (2020). One of the studies discussed there, reported that poorly controlled diabetes is strongly associated with higher prevalence, higher severity and faster progression of periodontitis in comparison with normoglycemic individuals, despite similar composition in subgingival biofilms (Nazir, 2017).

LGI has been proposed to be a relevant component of the interplay between periodontitis and diabetes. Severe PD in diabetic patients increases dramatically the risk of developing cardiovascular and renal diabetes-related complications in contrast to patients with no, mild, or moderate periodontitis. Periodontal therapy significantly reduces A1c hemoglobin levels, as well as circulating inflammatory mediators CRP, TNF, IL-6, and fibrinogen among others. Monocyte hyperactivity may be reversed in patients with diabetes mellitus by scaling and root planing inducing lower monocyte derived TNF-α, high sensitivity CRP, and sE-selectin levels (Cecoro et al., 2020).

Periodontal treatment may also play a role in enhancing insulin sensitivity and glycemic control via a reduction of both, periodontal inflammation and serum levels of cytokines and other inflammatory mediators (Baeza et al., 2020). Given that periodontal therapy induces lower hemoglobin A1c better than other glucose-lowering therapies, it may be envisioned that it can become an alternative or adjunctive therapy to enhance insulin sensitivity and glycemic control in diabetic patients with CP (Cecoro et al., 2020).

Leukocyte count has been reported as slightly elevated in subjects with CP. This increase directly correlates with the severity and extent of PD. Conversely, leukocyte counts decreased significantly after periodontal therapy. Along the same lines, IL-6 levels have also been consistently reported to be increased in proportion to the extent of PD (Cecoro et al., 2020). Also relevant in this context is the fact that PD triggers the release of polymorphonuclear leukocytes and, in combination with obesity, increases gene expression levels of IL-6, TNF-α, and CRP in the liver and adipose tissues. Though not properly metabolic diseases, cirrhosis and chronic kidney disease have been also associated with periodontal conditions (Grønkjær et al., 2018; Hickey et al., 2020) via similar mechanisms.

Obesity is correlated with periodontitis as discussed in the review by Cecoro et al. (2020). The authors reviewed the results of animal studies that analyzed the effects of periodontitis of the expression of pro-inflammatory cytokines in the liver and white adipose tissue (WAT) in Zucker rats. These results lead to conclude that in the obese rat model, periodontitis increased systemic LGI, hence driving increasing gene expression of hepatic levels of TNF and CRP and of IL-6 and CRP in adipose tissue (Table 3). In the lean rat model, periodontitis had little effect on pro-inflammatory cytokine gene expression in the liver and WAT (Cecoro et al., 2020). These authors also discussed the association between adiposity and LGI with tooth loss in men and women. Sex-specific differences in the incidence of PD and tooth loss seem to correlate with different phenotypes of obesity and LGI.

Periodontitis was found to drive chronic systemic LGI linked to atherosclerosis (Berlin-Broner et al., 2017). This association was found to be not significant in obese patients, likely due to a *masking* effect since adipose tissue produces inflammatory adipokines and cytokines, which may hide the purely periodontal disease effects on systemic LGI (Cecoro et al., 2020; Meisel et al., 2020). LGI is also argued to play a role as an intermediary state and a central hallmark of chronic diseases, such as obesity and diabetes mellitus. Fibrinogen levels and leukocyte counts point out to long-term associations with PD as quantified by probing depth and CAL, thereby reinforcing the association of PD and LGI with chronic inflammation and metabolic diseases such as diabetes, obesity, and metabolic syndrome (Meisel et al., 2020).

#### 2.1.3. Cardiovascular Disease

As is the case with other previously mentioned inflammation-related health conditions, consistent epidemiologic evidence has been found linking periodontitis with increased risk for cardiovascular diseases (CVDs) (Cardoso et al., 2018; Carrizales-Sepúlveda et al., 2018). Since vascular diseases are characterized by strong local and systemic inflammation, this is indeed unsurprising. In atherosclerosis, for instance, along with cholesterol debris accumulation on the artery walls, immune reactions via mediators such as cytokines are implicated in the pathogenesis of the disease (Cardoso et al., 2018). Inflammatory cytokines such as TNF-α, IL-1, and IL-6, among others, are secreted by infiltrating leukocytes or even by foam cells. The relevance of chronic infections in the atherosclerotic process, namely by inducing a systemic inflammatory state and autoimmunity, is also well established (Table 3).

Persistent microbial infections in the vessel wall are able to promote a pro-inflammatory environment. Infection can drive autoimmunity to vascular cells, thus initiating the atherosclerotic process (Hamilton et al., 2017). In this context, CP due to its sustained inflammatory nature correlates with an increased risk for cardiovascular disease. Bacteremia and systemic inflammation typical of CP are relevant factors leading to the onset of endothelial lesions, but also to the potentiation of inflammatory processes in the vascular wall (Leira et al., 2019b). Vascular inflammation is, in turn, modulated by pro-inflammatory cytokines such as TNF-α, IL-1, and IL-6, both in CP and in CVDs. It has even shown that a decrease in systemic inflammatory biomarkers secondary to periodontal treatment leads to beneficial features contributing to the reduction of cardiovascular risk (Preshaw et al., 2020). However, even if periodontal interventions result in a reduction of systemic inflammation and endothelial dysfunction in the short term, there is no significant evidence that these treatments actually prevent atherosclerotic vascular disease or are able to modify its outcomes (Cardoso et al., 2018).

Sustained periodontal inflammatory states will alter the count of circulating neutrophils due to increased bone marrow output or mobilization of the marginal granulocyte pool (Cecoro et al., 2020). Uptake of advanced glycation end products (AGEs) during PD is reported to drive pro-inflammatory signals that, in turn, trigger redox-sensitive transcription factors deemed responsible for hyper-permeability of the endothelial cell, VCAM-1 (vascular cell adhesion molecule 1) activation, chemotaxis and the release of TNF, IL-1, IL-6 into the bloodstream (Furutama et al., 2020). These circulating inflammatory mediators are able then to modify endothelial function, leading to impaired vasodilation and causing significant alterations in the vascular structure (Cecoro et al., 2020).

TNF-α and IL-6 in particular, are associated with diminishing nitric oxide production and endothelial dysfunction, both known precursor factors for atherosclerosis and CVDs. LGI triggered by PD may be detected via very low increases of CRP levels which, if sustained, may cause severe cumulative damage. As already discussed, LGI is considered to be one key mechanistic link between periodontitis and its comorbidities such as CVDs (Cecoro et al., 2020). The relationship between PD and CVDs has been traced back to factors such as transfer of periodontal bacteria to atheromatous plaques, change of lipid metabolism (Ferguson et al., 2020), endothelial dysfunction, shared genetic risk factors, as well as pro-inflammatory cytokine (especially IL-6 and TNF-α) spillover in the bloodstream coming from periodontal tissues. Severe PD, in particular, elevates the risks for acute myocardial infarction and stroke and it is known that periodontal treatment significantly reduces their incidence (Bui et al., 2019; Cecoro et al., 2020). It has been reported that chronic inflammation in PD has a significant impact on the long-term clinical outcomes in patients with atrial fibrillation (Im et al., 2018).

A meta-analysis that combined five cohort studies (86,092 patients) showed that individuals with PD had 1.14 times higher risk of developing coronary heart disease than controls, independent of known confounding factors. Case-control studies (1,423 subjects) pointed to an even greater odds ratio for developing coronary heart disease (2.22 times). Also the prevalence and incidence of CVDs resulted significantly increased in patients with PD. Furthermore, significant correlations between edentulousness and serum antibodies against *P. gingivalis* and *A. actinomycetemcomitans* with coronary heart disease were reported in a study including 1,163 men. The presence of bacterial DNA species in 42 atheromatous plaques retrieved by endarterectomy reported independently also supported the previous finding. The bacterial species most consistently reported were *P. gingivalis, A. actinomycetemcomitans, T. forsythia, F. nucleatum, Eikenella corrodens*, and *Campylobacter rectus*, respectively. Bacterial DNA (mostly of the same species and/or families) was found in human atherosclerotic plaques, further supporting the hypothesis that these oral pathogens migrate from the oral cavity to distant sites of the body (Bui et al., 2019). *Porphyromonas gingivalis* is able to evade innate immune detection via TLR-4 and enhance chronic inflammation in the vasculature. It can also drive platelet aggregation responsible for thrombus formation *in vivo* via secreted outer membrane vesicles. *Actinobacillus actinomycetemcomitans, T. forsythia, C. rectus, F. nucleatum, Prevotella intermedia*, and *T. denticola* in contrast, failed to aggregate platelets when tested for aggregation activity, suggesting that within these common oral pathogens, only *P. gingivalis* expresses platelet-aggregating virulence factors (Bui et al., 2019).

Animal models of atherosclerosis studying hyperlipidemic mice infected with *P. gingivalis and T. denticola* showed that infection with these bacteria is associated with alveolar bone loss and aortic atherosclerosis. Secondary to oral infection, *P. gingivalis and T. denticola* were able to trigger systemic immune responses, as it was the case in human subjects with a naturally occurring infection, bacterial genomic DNA was found in the oral epithelium, aorta and within systemic organs in these animal models. Some studies have thus suggested that there may be an association between PD and CVD, nevertheless the impact of oral infection in cardiovascular diseases has remained unclear (Bui et al., 2019).

CP is known to affect the vascular epithelium leading to arterial stenosis. In this regard, an association between periodontitis and peripheral arterial disease (PAD) has been found, leading to a reduction of arterial flow. A decreased tooth count, as well as worsened oral and periodontal conditions have been reported, in PAD patients, concomitant with other effects of enhanced systemic inflammation. This becomes especially relevant in view that PAD is strongly associated with higher morbimortality in CVDs, comprising more than half of the patients with coronary artery and cerebrovascular diseases (Aoyama et al., 2017). In this regard, potential mechanisms of molecular inflammation have been proposed to link PD and PAD. In brief, it has been advanced that this may start with periodontitis. PD is able to induce frequent bacteremia, which in turn may cause damage to endothelial tissue, bacterially-induced LPS production and release leading to chronic activation of circulating monocytes. Activated monocytes signals promote atherogenesis that, in combination with increased pro-inflammatory factors, are able to trigger PAD (Aoyama et al., 2017; Bunte and Beikler, 2019).

#### 2.1.4. Neurological Diseases

CP has been linked not only to systemic inflammation, but also to neuroinflammation and other forms of neurological damage. For instance, it has been known for some time that PD is more prevalent in patients with Parkinson's disease (Kaur et al., 2016). In particular, those periodontitis patients showed a significantly higher rate of 4 mm deep periodontal pockets when compared to patients without such neurodegenerative disease (98.6 vs. 43.5%, respectively) (Cecoro et al., 2020). The molecular basis of cognitive decline has been traced back to the formation of synaptotoxic β-amyloid plaques and hyperphosphorylated τ proteins in the regions of the brain associated with advanced cognition. As it was discussed in relation to diabetes, neurological conditions such as Parkinson's and Alzheimer's diseases and periodontitis also present bidirectional relationships (Ganesh et al., 2017; Leblhuber et al., 2020; Sansores-España et al., 2020). Hence, establishing causality may be tricky since correlation may be attributed not only to PD-induced neuroinflammation associated with neurological disease, but also to motor disability and cognitive impairment diminishing the efficacy of oral hygiene thus leading to periodontitis (Diomede et al., 2017; Bui et al., 2019). It was recently shown indeed, that individuals with brain injury present higher prevalence of poor oral health parameters as well as generalized CP (Bui et al., 2019). On the other hand, it has been argued, for instance, that pro-inflammatory cytokines released into the systemic bloodstream via the ulcerated periodontal pockets may actually be able to weaken the blood brain barrier, thus allowing access to the cerebral regions, triggering neuronal damaging signaling cascades secondary to microglial cell activation (Cecoro et al., 2020; Gil Montoya et al., 2020).

Activated glial cells are able to produce significant levels of inflammatory cytokines similar to the ones associated with Alzheimer's disease (AD). Direct damage caused by β-amyloid plaques and τ aggregates, is further aggravated by the innate immune response signals to purge these aggregates outside the brain, thus increasing neurodegeneration (Leira et al., 2020a). Increased levels of pro-inflammatory cytokines have actually been detected in elderly patients with AD and PD. Immune signaling assays using different anti-inflammatory drugs and cytokines reinforced the hypothesis associating inflammation as a major driver of neurodegeneration in AD. These studies have also pointed to nasal nonsteroidal anti-inflammatory drugs (NSAIDs), as potentially effective therapies to slow-down the onset of AD. Furthermore, the IL-1 receptor antagonist and immunosuppressive cytokines are able to protect the brain from additional damage thus preventing AD progression (Bui et al., 2019). LPS harvested from periodontal pathogens *P. gingivalis* and *T. denticola* was isolated from peri-postmortem human brains with AD, indicating that these pathogens are relevant for the development of brain inflammation in relation to Alzheimer's disease (Bui et al., 2019).

Hence, both inflammatory mediators, as well as some periodontal pathogens are able to invade the brain by crossing the brain-blood barrier. This fact was further confirmed in animal models (Bui et al., 2019). In consequence, periodontal infection and its secondary immune responses may have effects beyond the blood-brain barrier, potentially unleashing neurological disorders. Wang and collaborators, for instance, have extensively discussed the molecular and physiological mechanisms linking CP and cognitive decline (Wang et al., 2019).

Depression—which is more properly described as a psychiatric ailment than a neurological disease—has also been associated with system-wide inflammatory processes and chronic periodontitis (Hashioka et al., 2018) involving the so called neuroinflammation processes (Table 3; Teixeira et al., 2017; Furutama et al., 2020).

#### 2.1.5. Chronic Respiratory Diseases

Since the oral cavity is fully connected to the respiratory airway, it is unsurprising that infectious and inflammatory conditions are shared between these two anatomic locations. The oral cavity is a known reservoir for pulmonary infections. Indeed, CP has been directly associated with risk of asthma, pneumonia, or chronic obstructive pulmonary disease (COPD) (Moghadam et al., 2017; Gomes-Filho et al., 2020). It is argued that oral bacteria present in the dental plaque may leak into saliva then move down into the lower respiratory tract and the lungs leading to respiratory infection (Table 3; Spiropoulou et al., 2019).

As in previously discussed conditions, not only pathogens, but also cytokines and enzymes, resulting from periodontal inflammation can flow into the lungs (Fabri, 2020). Once there, these molecules may signal cascades stimulating local inflammatory processes in advance of further pathogenic colonization (Cardoso et al., 2018). Among the periodontal pathogens that have been related to this complex comorbidity conditions are *A. actinomycetemcomitans, Actinomyces israelii, Capnocytophaga* spp., *C. pneumoniae, E. corrodens, F. nucleatum, Fusobacterium necrophorum, P. gingivalis, P. intermedia*, and *Streptococcus constellatus*. *Fusobacterium nucleatum* and *Fusobacterium necrophorum* were indeed associated with a different, but related condition starting with pharyngitis, later developing into a full respiratory tract infection called Lemierre's syndrome (Akkaoui et al., 2020). Also a cross-sectional study in a student cohort reported acute sore throat associated to *F. necrophorum* in 20.5% of subjects and 9.4% of asymptomatic individuals. This research points out to *Fusobacterium* as a potential lung pathogen to be considered when investigating airway complications (Bui et al., 2019).

The relationship between oral infection, microbial mobility and infection/inflammation of the respiratory airway has been confirmed by animal models. For instance, in a mouse model of intratracheal infection, it was revealed that *P. gingivalis* led to persistent inflammatory responses in the lungs via a joint mechanism of cell recruitment and pro-inflammatory cytokine production. These results also correlated with results in humans, a study with 40 subjects subject to orotracheal intubation showed increased abundance of *A. actinomycetemcomitans, P. gingivalis* and *T. forsythia* both in toothed and toothless subjects, which led the authors to hypothesize that the oral environment, even in the absence of teeth, becomes a favorable environment for pathogenic bacterial accumulation (Bui et al., 2019).

#### 2.1.6. Inflammatory and Autoimmune Diseases

The comorbidity spectrum of periodontal disease is, as we have seen, quite broad. A number of conditions, related to the presence and migration of infectious periodontal pathogens, to the underlying immune response to these, and to the acute and chronic inflammatory processes unleashed by these events are able to affect the organisms in a plethora of (often disparate) manners (Berlin-Broner et al., 2017; Hickey et al., 2020). Let us consider some of these additional disease conditions. Particularly relevant is the case of autoimmune conditions. We have already discussed the interplay between innate and adaptive immunity (see Figure 2). Any process disturbing this delicate balance, for instance, chronic infections, may bring important consequences. In particular, it may trigger autoimmune responses. Such responses are known to be major underlying factors in the onset and progression of the most common immune-mediated inflammatory diseases, such as rheumatoid arthritis, systemic lupus erythematosus, and inflammatory bowel diseases (Piras et al., 2017; Bunte and Beikler, 2019).

In the case of rheumatoid arthritis (RA), it has been shown that RA patients present higher bacterial loads, concomitant with an increased abundance of pathogenic species, and a diversified and dysbiotic oral microbiota, following patterns similar to those associated with PD (Table 3) with an increased CAL (de Molon et al., 2019). Peptide citrullination mediated by peptidylarginine deiminase is considered a central player in RA. *Porphyromonas gingivalis* has been shown to express peptidylarginine deiminase, a molecular clue that can represent a direct biological common factor between PD and RA. In agreement to this, recent studies have strengthened the hypothesis that PD is a risk factor for RA development: correlates show that greater severity of RA (swollen joints), increased erythrocyte sedimentation rate (ESR) and CRP typical of RA, can be associated with severe instances of periodontal bone resorption (de Molon et al., 2019; Bartold and Lopez-Oliva, 2020).

Synovitis, the inflammation of the synovial membrane in joints, is actually able to induce the clinical signs and symptoms of RA. In synovitis, a complex signaling network comprising immune cells and cytokines is at play. By the action of these intertwined cascades, immune cell recruitment and infiltration occurs causing the synovial membrane to vascularize and become infiltrated with fibroblasts, macrophages, T- and B- cells, plasma cells, mast cells, dendritic cells, and neutrophils. Mixed signals include increased TNF in the synovial fluid, able to trigger IL-1 production, unleashing B and T cell activation and a pro-inflammatory cascade mediated by Th-17 as well as dendritic cell induction destroying the cartilage and the articular bone. Crosstalk of IL-1 and IL-17 with TNF drives T cell and immature dendritic cell chemotaxis, as well as stimulation of fibroblasts and epithelial cells to secrete IL-6, IL-8, PGE2 (prostaglandin E2), and neutrophil chemoattractants, further increasing inflammation (Ceccarelli et al., 2019; Schulz et al., 2019). The links between synovitis and CP are further established by the fact that an IL-22 associated mechanism increased synovial inflammation in RA joints and clinical attachment loss in CP patients, mimicking IL-22 pro-inflammatory function in psoriasis (Ancuta et al., 2017; Bunte and Beikler, 2019).

It has been hypothesized that the periodontal microbiota may be involved in RA progression via increasing epithelial and mucosal permeability, enhancing the loss of immune tolerance to components of the microbiota, as well as facilitating immune cell tracking to the joints. Along the same lines, periodontal pathogens become able to reach the blood circulation as a consequence of frequent and low intensity bacteremia, perhaps induced by chewing or tooth brushing. This is further supported by the fact that DNA of *P. gingivalis, T. denticola, P. intermedia, Prevotella nigrescens, T. forsythia*, and *F. nucleatum* and other periodontal microbes has been detected in the synovial fluid of patients with RA. Furthermore, elevated antibody titers against *T. forsythia, P. intermedia*, and *P. gingivalis* have been detected in the serum and synovial fluid of RA patients (Gómez-Bañuelos et al., 2019). RA and PD share a number of commonalities, including shared genetic susceptibility origins that may be associated with a common epitope (SE)-coding HLA-DRB1 allele—associated with bone erosions in RA, as well as alveolar bone destruction during PD—, microbial abundance patterns (*P. gingivalis bacteria*, microbial dysbiosis at distant sites, such as the gut microbiome Lourenςo et al., 2018; Li et al., 2020, and the role of citrullination, and anti-citrullinated protein antibodies), as well as intrinsic inflammatory response features (cytokines and immune inflammatory responses to the Th17 profile) (de Molon et al., 2019).

Another immune-mediated inflammatory condition that has been associated with PD is systemic lupus erythematosus (SLE), in this condition circulating pro-inflammatory cytokines are also upregulated in patients producing an LGI status (Cecoro et al., 2020). The interaction of Th17 cells, Tregs, CD8+ cells, B cell subsets, and innate immune system cells, reduced IL-2 and an increased IL-17 production (Bunte and Beikler, 2019). The microbiome composition disrupted in PD affects the levels of a wide range of cytokines involved in SLE (Pessoa et al., 2019).

Inflammatory bowel diseases (IBD) constitutes a family of immune-mediated inflammatory diseases of the gastrointestinal tract, including ulcerative colitis, Crohn's disease and other less common syndrome-like diseases. IBDs are known to originate from the confluency of a dysbiotic intestinal microbiome with a number of lifestyle, genetic and environmental risk factors, including smoking and an *inflammatory* diet (Lira-Junior and Figueredo, 2016). It has been argued that disease onset happens via activation of inflammatory pathways leading to the disruption of the epithelial barrier integrity in genetically susceptible individuals (Piras et al., 2017). A number of cytokine cascades such as the ones initiated by IL-23 and IL-17 have also been reported in connection with IBD. As it may result evident at this point, microbial dysbiosis and the presence of cascades of pro-inflammatory cytokines and even risk factors are shared between IBD and CP (Bunte and Beikler, 2019). This is also the case of other inflammatory/autoimmune conditions such as Sjögren syndrome, a condition presenting diffuse lymphocyte infiltration into exocrine glands causing xerostomia and ocular dryness. Increased secretion and expression of IL-17 as well as an abundant presence of IL-1β, TGF-β, IL-6, and IL-23 in tissues affected by the syndrome, demonstrate the significance of Th17 cells and IL-17 in the immunopathogenesis of this disease (Katsifis et al., 2009).

We have already mentioned that both, oral pathogens and immune response molecules are able to migrate to the bloodstream and that vascular vessels communicate also with the oral cavities. Hence, it is unsurprising that there are associations between PD and hematological diseases (Acharya et al., 2019; Hajishengallis, 2020). In the case of leukemia, it is known that some of the oral manifestations of this disease are mucosal bleeding, ulceration, petechiae, as well as gingival enlargement (either diffuse or localized). Gingival infiltration by leukemic cells is present commonly in both, acute monocytic leukemia and acute myelomonocytic leukemia, and has been also documented in PD (Wang et al., 2020). Impaired immune function can lead to various secondary oral complications, such as candidiasis, herpes simplex virus infection, and periodontal bone loss, which also correlates with PD symptoms and comorbidities (Chi et al., 2010). In the case of thrombocytopenia, diagnosis often starts with signs of oral lesion development. Due to platelet disruption, minor trauma to the oral mucosa during everyday tasks like chewing or swallowing may result in hemorrhagic lesions: petechiae, purpura, ecchymosis, hemorrhagic bullae, and hematoma formation among others (Chi et al., 2010). As in previous conditions discussed, the relationship between PD and these conditions seems to be bi-directional.

#### 2.1.7. Obstetric Complications

Pathogen migration and systemic inflammation associated with CP, has been documented to be also relevant in the context of obstetric complications: Along pregnancy, vascular permeability in the gingival tissues is increased, triggered by hormonal changes (Bartold and Van Dyke, 2019). Enhanced permeability then eases the diffusion of pathogenic microorganisms and their products into the bloodstream, even reaching the placenta. Once these pathogenic agents are there, they may drive immune and inflammatory responses inducing augmented secretion of pro-inflammatory cytokines potentially affecting the fetal tissues (Zi et al., 2015). Fetal inflammation can in turn lead to the premature rupture of membranes, causing uterine contractions, augmenting the risk of miscarriage or premature delivery (Ye et al., 2020). Complications during pregnancy have also been documented to be associated with CP and inflammation: Mohr et al. (2019) reported on the influence of systemic inflammation and preterm prelabor rupture of membranes in pregnant women with PD. Similar effects have indeed been traced back to periodontal bacterial infections (Ye et al., 2020).

The presence of periodontal pathogenic microorganisms in the amniotic fluid in women with periodontitis has been also associated with the severity of periodontitis related to the risk of preterm birth (Cardoso et al., 2018). *Actinobacillus actinomycetemcomitans, E. corrodens, P. gingivalis*, and *T. denticola* have been associated with gestational hypertensive disorders. Likewise, *Bergeyella* sp., *Capnocytophaga* spp., *E. corrodens, Parvimonas micra, P. gingivalis, T. forsythia*, and *T. denticola* have been detected in certain women with low birth weight (Table 3; Bobetsis et al., 2020).

In this regard, at least two mechanisms have been proposed relating periodontal health status and adverse pregnancy outcomes. The first one argues that oral periodontal pathogens are able to translocate from infectious oral cavities all the way down to the placenta, potentially reaching the intra-amniotic fluid and fetal circulation. The second hypothesis states that the PD-driven systemic endotoxin or inflammatory mediator dissemination is able to modify fetal development even leading to spontaneous miscarriage. A recent study states that *F. nucleatum* is the most common among the periodontal pathogens found in placental and fetal tissues (Bobetsis et al., 2020; Ye et al., 2020). This has led to hypothesize that *F. nucleatum* is able to translocate from the maternal mouth to the uterus, due to immune response weakening of infectious origins (Bui et al., 2019).

Additionally, research in animal models has shown that *P. gingivalis* can have a negative impact on pregnancy. It was observed that LPS from *P. gingivalis* leads to placental and fetal growth restriction and resorption in rats. Anti-*P. gingivalis* antibodies were also able to induce fetal loss when passively administered to mice. There is still scarce evidence regarding the role of innate immune receptors during pregnancy. While it has been reported that the placenta can express TLRs during normal pregnancy. PD or even abundance of periodontal pathogens such as *T. denticola* and *P. gingivalis* have been associated with an increase in the expression of TLRs, thus suggesting enhanced innate immune responses (Bui et al., 2019). The role that hormones will be playing in these phenomena is still not understood. However, the significant associations of periodontal inflammation, systemic inflammation and duration of menopausal years in postmenopausal women indicate that hormone regulation and unresolved inflammation in CP may be related (Sharma et al., 2018).

#### 2.1.8. Systemic Bone Loss

Other conditions, in particular those affecting tissue and cell matrix integrity, are also associated with chronic inflammatory scenarios typical of unresolved PD. This is the case of osteoporosis (Wang and McCauley, 2016). Recent studies have suggested a bidirectional relationship between PD and systemic bone loss (Penoni et al., 2019). As it was mentioned, osteoclast regulation is affected by a number of cytokines and second messengers produced during the course of CP, such as IL-17/Th17 pathway activity (Bernal et al., 2018; Duka et al., 2019). Also, the potential explanation for the influence of periodontitis on systemic bone density is related to moderate and severe periodontal infection. After adjusting for age, gender, and comorbid systemic disorders, Mau and coworkers showed that patients with chronic periodontitis present an increased risk of osteoporosis over a 6-year follow-up period (2.72 and 1.66/1,000 of periodontitis cohort vs controls subjects, respectively) (Mau et al., 2017).

The association of PD and osteoporosis has been thoroughly studied in recent years, however, the underlying mechanism of how PD affects bone metabolism remains unclear (Mau et al., 2017; Penoni et al., 2019). Conversely, it has been shown that anti-inflammatory therapy such as inhibition of angiotensin II receptor I, is able to prevent both inflammation and bone loss, in periodontitis (Li et al., 2019a). Other anti-inflammatory and immune regulatory processes are known to have effects on bone loss (Aral et al., 2018; Sima et al., 2019; Paula-Silva et al., 2020). EGFR (epidermal growth factor receptor) signaling has been found to downregulate the αvβ6 integrin, thus promoting periodontal inflammation and bone loss (Bi et al., 2020). The protective effects of 6-Formylindolo[3,2-b]carbazole (FICZ) and the aryl hydrocarbon receptor signaling pathway on alveolar bone loss and inflammation have been recently discussed in the context of experimental periodontitis (Huang et al., 2019).

#### 2.1.9. COVID-19, Inflammation, and Periodontitis

The recent, still ongoing COVID-19 pandemic has significantly altered many of our notions and perceptions of chronic and inflammatory conditions. This is also the case with periodontal inflammation. As we have discussed extensively, the roots of the pathophysiology of CP lie, to a large proportion, on a disproportionate cytokine response. As it is the case, the so-called *cytokine storms* also contribute to a large extent in COVID-19 related complications and mortality (Tang et al., 2020; Hu et al., 2021). COVID-19 and CP share similar cytokine storm profiles, characterized by elevated levels of IL-1β, IL-7, IL-10, IL-17, IL-2, IL-8, IL-9, IFN-γ, TGF-β, as well as some metalloproteinases (Sahni and Gupta, 2020). As previously presented, CP often involves Th17 inflammatory responses that are, indeed, one of the determinant drivers of cytokine storming (Bunte and Beikler, 2019).

These facts are particularly relevant in view that the age group that has been more severely affected by COVID-19 morbi-mortality, older people, is also the group that are more commonly affected by CP (Ebersole et al., 2016). Aside from cytokine storms, multi-infection may occur since periodontal pathogens can be inoculated during the course of endotracheal intubation or even aspirated into the lower respiratory tract. This may be an aggravating factor, as it has been documented that periodontal bacteria have the potential to aggravate age-related senescent cell accumulation, thus improving the attachment and replication of SARS-CoV2 (Aquino-Martinez and Hernández-Vigueras, 2021; Elisetti, 2021). We should also not disregard that hypertension is a common CP comorbidity that has also been a documented risk factor for aggravated COVID-19 (Botros et al., 2020). Patients with severe COVID-19, associated with acute severe respiratory distress, are known to present elevated levels of IL-6, C reactive protein, and ferritin signs of unresolved hyper-inflammation. These factors have been started to be investigated in regulated case-control studies that have found that after confounding factor adjustment, PD was associated with COVID-19 complications such as death (OR = 8.81, 95% CI 1.00–77.7), ICU admission (OR = 3.54, 95% CI 1.39–9.05), and the need for assisted ventilation (OR = 4.57, 95% CI 1.19–17.4) (Marouf et al., 2021).

There is growing evidence suggesting a relevant role of the oral mucosa in both transmission and pathogenicity of SARS-CoV2, especially in view of large levels of the ACE2 receptor present in the oral cavities (Iranmanesh et al., 2021). Aside from infectious origins and unresolved inflammation, COVID-19 and PD, also share a number of risk factors and, interestingly, an association with the production of Neutrophil Extracellular Trap (NET). NET over-production is able to induce *NETosis*, a not-so-common form of cell death, by creating an entanglement of chromatin decondensates by primed neutrophils leading to tissue degradation. As it turns out both CP and COVID-19 may present tissue damage by NETosis (Gupta and Sahni, 2020).

### 2.2. Periodontal Inflammation: Biomarkers and Diagnostics

Genetic markers have been associated with different outcomes and conditions related to PD, CP and their comorbidities. For instance, regarding the susceptibility to develop nonalcoholic fatty liver disease, Akinkugbe et al. (2017) have documented to be associated with a number of genetic markers of inflammation. The presence and abundance of certain microorganisms have also been used as biomarkers for the severity of the inflammatory periodontal condition (Bui et al., 2019).

Immune markers have been associated with the effects of PD and systemic inflammation in type 2 diabetes (Singhal et al., 2016). Adiponectin levels have been recently discussed to correlate with periodontal inflammation and diabetes (Purnamasari et al., 2019), while resistin has been argued to be a potential CP biomarker (Akram et al., 2017) and chemerin levels in gingival crevicular fluid and tears are potential biomarkers of inflammation in chronic periodontitis and type-2 diabetes mellitus (Patnaik et al., 2017).

Tan and collaborators recently reported that an increase in serum and salivary neutrophil gelatinase-associated lipocalin is positively correlated with periodontal inflammation (Tan et al., 2020) though further independent validation trials are currently being carried out. Levels of MMP-12 and of the damage-associated molecular pattern (DAMP) molecule S100s in saliva have been found to be effective markers of PD progression (Holmström et al., 2019). MMP-8 has been also mentioned as a potential point-of-care test biomarker for progression of periodontal inflammation (Lorenz et al., 2017). A similar association has been reported in connection with increased serum calcitonin-related peptides in PD patients suffering migraines (Leira et al., 2019a).

Exosomal nucleic acids have been extensively studied recently in search of reliable biomarkers. High throughput assays, though still in the early stages, are becoming promising techniques to find specific biomarkers in PD, such is the case of proteomics studies (Bostanci and Bao, 2017). Genetic assays are also under development. By using these techniques it was possible to find out that exosomal PD-L1 RNA in saliva may constitute an effective early biomarker of PD (Yu et al., 2019).

### 2.3. Periodontal Inflammation: Drugs and Therapeutics

Given the fact that sustained, unresolved inflammation is one of the key elements involved in periodontitis-associated morbidity, it is not surprising that many of the therapeutic approaches to treat the conditions are related to controlling such extended inflammation. Such is the case of statins, for instance. Petit et al. (2019) recently summarized a number of pharmacological applications of statins to treat chronic periodontitis. Oleuropein, a drug derived as a natural product, has been shown to have important therapeutic effects on alveolar bone loss, inflammation, and apoptosis in experimental periodontitis assays (Taskan et al., 2019). The use of inflammation *resolvers* rather than inflammation *inhibitors* has started to gain acceptance as a relevant design in therapeutic approaches to treat PD (Sima and Van Dyke, 2016; Van Dyke, 2020). Resolvin, for instance, has been used as a coadjuvant therapy to treat atherosclerosis in PD patients (Hamilton et al., 2017). The use of colchicine, has also shown to have important effects vs. gingival inflammation, apoptosis, and alveolar bone loss (Aral et al., 2018). A similar finding was found for 5-lipoxygenase therapy in polymicrobial apical periodontitis (Paula-Silva et al., 2020), and in an experimental model for rosuvastatin therapy (Kırzıoğlu et al., 2017). Along similar lines, the inhibitory effects of panduratin A were documented on periodontitis-induced inflammation and osteoclastogenesis via *in vitro* inhibition of MAPK (intracellular enzymes involved in the response to stimuli such as inflammatory cytokines) pathways (Zhang and Li, 2015; Kim et al., 2018).

Reducing chronic inflammation is the first line of treatment for patients with CP and related ailments. To this end, Hong and collaborators developed a randomized, double-blind, placebo-controlled, multicenter study to assess the effects of fixed-dose combinations of vitamin C, vitamin E, lysozyme, and carbazochrome on gingival inflammation in patients with CP (Hong et al., 2019). Host modulation therapies (Bartold and Van Dyke, 2017; Hajishengallis et al., 2020) combining anti-inflammatory and antioxidant agents have shown promising effects to treat PD in clinical trials (Sulijaya et al., 2019). Other biologics used as novel therapeutic strategies involve molecules able to trigger myeloid cell receptors (Rudick et al., 2017) since these cells are known players in pathogenic inflammation (Peddis et al., 2019). The novel anti-inflammatory drug tetramethylpyrazine has been recently found to reduce inflammation levels as well as LPS-mediated apoptosis in human periodontal ligament cells by a mechanism involving the downregulation of the small non-coding RNA miR-302b (Duan et al., 2020).

Aside from the effects of pharmacological therapy in CP and related conditions, it is important to consider the potential effects of drugs used to treat other diseases (and even of polypharmacy) on the inflammation and immune responses in PD. Gusman et al. (2019), for instance, have found that certain antineoplastic agents are able to exacerbate periodontal inflammation and aggravate periodontitis in experimental models.

### 2.4. Periodontal Inflammation: Diet and Lifestyle

Diet and lifestyle are also known to be relevant players in the onset and development of chronic periodontal disease and its many comorbidities. In a recent study, Dal-Fabbro and collaborators found a clear relationship between chronic alcohol consumption and sustained inflammation in relation to osteoclastogenesis in apical periodontitis (Dal-Fabbro et al., 2019). Smoking has been long associated with respiratory conditions such as COPD, emphysema, as well as lung and head and neck cancers. Recent evidence, however, has established a link between smoking and periodontitis leading to respiratory airway fibrosis accelerating COPD (Spiropoulou et al., 2019). Inflammation has been reported to have synergistic effects with nicotine consumption in PD via upregulating α7 nAChR via phosphorylated GSK-3β (Zhou et al., 2020). On the other hand, it has been documented—on a randomized controlled pilot study—that an optimized diet may reduce gingival and periodontal inflammation in humans (Woelber et al., 2017).

In the case of diet, several studies have revealed an association between dietary factors and periodontitis (Al-Zahrani et al., 2005; Alhassani et al., 2021). For instance, it has been argued that a so-called *industrialized western diet* rich in processed carbohydrates like sugar, white flour, and processed fatty acids like trans fats and poor in micronutrients promotes gingival and periodontal inflammation (Woelber and Tennert, 2020). A cross-sectional study in 10,000 participants in the NHANES cohort, on the other hand, revealed the effects of a *protective diet* (Wright et al., 2020). In some instances, associations have been also revealed with factors such as the composition of the oral microbiota (Jockel-Schneider et al., 2021; Li et al., 2021). Diet has also been related to inflammation in periodontitis (Woelber et al., 2017; Kotsakis et al., 2018; Botelho et al., 2021; Rowińska et al., 2021) and other conditions as disparate as cancer (Deng et al., 2017; De Almeida et al., 2019), diabetes (Prana et al., 2019; Jafaripour et al., 2020), cardiovascular diseases (Razquin and Martinez-Gonzalez, 2019; Kovell et al., 2020), Alzheimer's disease (McGrattan et al., 2019), depression (Koopman and El Aidy, 2017), and even COVID-19 (Iddir et al., 2020).

## 3. Concluding Remarks

A growing body of evidence is accumulating supporting the direct and indirect impact of periodontal pathogens on systemic health. Epidemiological, clinical, and experimental studies have revealed the often overlooked relationship between bacteremia or inflammation due to periodontal disease and systemic disease (Bui et al., 2019). A deeper understanding of the interplay between pro-inflammatory and suppressor or anti-inflammatory responses, able to account for internal and external environmental factors, is doubtless needed in-route to improved therapeutic interventions with the potential to modulate host responses in CP, as well as in other chronic inflammatory diseases (Cardoso et al., 2018; Cecoro et al., 2020).

Here we have comprehensively summarized the recent corpus of literature related to how periodontitis goes from a seemingly mild, localized infectious disease, to a potentially life-threatening, chronic generalized hyper-inflammation condition. Some of the biological issues related to such progression and leading to its many comorbidities were also touched upon. The main medical conditions that may often become complicated by co-existing with chronic periodontitis were presented and discussed in the context of systemic inflammation, microbial dysbiosis, and the rise of common and intertwined risk factors. Our ultimate goal is to continue opening a dialogue on the many facets of periodontal disease, that may establish the foundations to study, diagnose, and treat this complex systemic condition under an integrative framework.

## Author Contributions

EH-L conceived the project. EH-L and MM-G performed research and wrote the manuscript. Both authors reviewed and approved the manuscript.

## Funding

This work was supported by CONACYT (Grant No. 285544/2016 Ciencia Básica and Grant No. 2115 Fronteras de la Ciencia), as well as by federal funding from the National Institute of Genomic Medicine (Mexico). Additional support has been granted by the National Laboratory of Complexity Sciences (Grant No. 232647/2014 CONACYT). EH-L acknowledges additional support from the 2016 Marcos Moshinsky Fellowship in the Physical Sciences.

## Conflict of Interest

The authors declare that the research was conducted in the absence of any commercial or financial relationships that could be construed as a potential conflict of interest.

## Publisher's Note

All claims expressed in this article are solely those of the authors and do not necessarily represent those of their affiliated organizations, or those of the publisher, the editors and the reviewers. Any product that may be evaluated in this article, or claim that may be made by its manufacturer, is not guaranteed or endorsed by the publisher.

## References

[B1] Aarabi G. Zeller T. Seedorf H. Reissmann D. Heydecke G. Schaefer A.. (2017). Genetic susceptibility contributing to periodontal and cardiovascular disease. J. Dent. Res. 96, 610–617. 10.1177/0022034517699786 28530468

[B2] Acharya A. B. Shetty I. P. Jain S. Padakannaya I. Acharya S. Shettar L.. (2019). Neutrophil-to-lymphocyte ratio and platelet-to-lymphocyte ratio in chronic periodontitis before and after nonsurgical therapy. J. Indian Soc. Periodontol. 23:419. 10.4103/jisp.jisp_622_18 31543614 PMC6737853

[B3] Akinkugbe A. Avery C. Barritt A. Cole S. Lerch M. Mayerle J.. (2017). Do genetic markers of inflammation modify the relationship between periodontitis and nonalcoholic fatty liver disease? Findings from the ship study. J. Dent. Res. 96, 1392–1399. 10.1177/0022034517720924 28732187 PMC5652859

[B4] Akkaoui J. Yamada C. Duarte C. Ho A. Vardar-Sengul S. Kawai T.. (2020). Contribution of porphyromonas gingivalis lipopolysaccharide to experimental periodontitis in relation to aging. GeroScience 43, 367–376. 10.1007/s11357-020-00258-1 32851571 PMC8050187

[B5] Akram Z. Rahim Z. H. A. Taiyeb-Ali T. B. Shahdan M. S. A. Baharuddin N. A. Vaithilingam R. D.. (2017). Resistin as potential biomarker for chronic periodontitis: a systematic review and meta-analysis. Arch. Oral Biol. 73, 311–320. 10.1016/j.archoralbio.2016.08.016 27567495

[B6] Alhassani A. A. Hu F. B. Li Y. Rosner B. A. Willett W. C. Joshipura K. J. (2021). The associations between major dietary patterns and risk of periodontitis. J. Clin. Periodontol. 48, 2–14. 10.1111/jcpe.13380 33020936

[B7] Alrayyes S. Hart T. C. (2011). Periodontal disease in children. Dis.-a-Month 57, 184–191. 10.1016/j.disamonth.2011.03.004 21569881

[B8] Al-Zahrani M. S. Bissada N. F. Borawski E. A. (2005). Diet and periodontitis. J. Int. Acad. Periodontol. 7, 21–26. 15736892

[B9] Ancuta C. Ancuta E. Chirieac R. Anton C. Surlari Z. Iordache C. (2017). Tnf inhibitors and periodontal inflammation in psoriatic arthritis. Rev. Chim. 68, 1914–1918. 10.37358/RC.17.8.5790

[B10] Aoyama N. Suzuki J.-I. Kobayashi N. Hanatani T. Ashigaki N. Yoshida A.. (2017). Periodontitis deteriorates peripheral arterial disease in Japanese population via enhanced systemic inflammation. Heart Vessels 32, 1314–1319. 10.1007/s00380-017-1003-6 28567552

[B11] Aquino-Martinez R. Hernández-Vigueras S. (2021). Severe covid-19 lung infection in older people and periodontitis. J. Clin. Med. 10:279. 10.3390/jcm10020279 33466585 PMC7828740

[B12] Aral C. A. Aral K. Yay A. Özçoban Ö. Berdeli A. Saraymen R. (2018). Effects of colchicine on gingival inflammation, apoptosis, and alveolar bone loss in experimental periodontitis. J. Periodontol. 89, 577–585. 10.1002/JPER.17-0359 29520818

[B13] Ari G. Cherukuri S. Namasivayam A. (2016). Epigenetics and periodontitis: a contemporary review. J. Clin. Diagn. Res. 10:ZE07. 10.7860/JCDR/2016/21025.8864 28050521 PMC5198474

[B14] Badran Z. Gaudin A. Struillou X. Amador G. Soueidan A. (2020). Periodontal pockets: a potential reservoir for sars-cov-2? Med. Hypothes. 143:109907. 10.1016/j.mehy.2020.109907 32504927 PMC7833827

[B15] Baeza M. Morales A. Cisterna C. Cavalla F. Jara G. Isamitt Y.. (2020). Effect of periodontal treatment in patients with periodontitis and diabetes: systematic review and meta-analysis. J. Appl. Oral Sci. 28:e20190248. 10.1590/1678-7757-2019-0248 31939522 PMC6919200

[B16] Balta M. G. Loos B. G. Nicu E. A. (2017). Emerging concepts in the resolution of periodontal inflammation: a role for resolvin e1. Front. Immunol. 8:1682. 10.3389/fimmu.2017.01682 29312286 PMC5735081

[B17] Bartold P. M. Lopez-Oliva I. (2020). Periodontitis and rheumatoid arthritis: an update 2012-2017. Periodontology 2000 83, 189–212. 10.1111/prd.12300 32385878

[B18] Bartold P. M. Van Dyke T. E. (2017). Host modulation: controlling the inflammation to control the infection. Periodontology 2000 75, 317–329. 10.1111/prd.12169 28758299

[B19] Bartold P. M. Van Dyke T. E. (2019). An appraisal of the role of specific bacteria in the initial pathogenesis of periodontitis. J. Clin. Periodontal. 46, 6–11. 10.1111/jcpe.13046 30556922 PMC6357965

[B20] Behm C. Blufstein A. Gahn J. Noroozkhan N. Moritz A. Rausch-Fan X.. (2019). Soluble cd14 enhances the response of periodontal ligament stem cells to toll-like receptor 2 agonists. Mediat. Inflamm. 2019:8127301. 10.1155/2019/8127301 31178663 PMC6507176

[B21] Belibasakis G. N. Kast J. I. Thurnheer T. Akdis C. A. Bostanci N. (2015). The expression of gingival epithelial junctions in response to subgingival biofilms. Virulence 6, 704–709. 10.1080/21505594.2015.1081731 26305580 PMC4720238

[B22] Berlin-Broner Y. Febbraio M. Levin L. (2017). Apical periodontitis and atherosclerosis: is there a link? Review of the literature and potential mechanism of linkage. Quintess. Int. 48, 527–553. 10.3290/j.qi.a38162 28462408

[B23] Bernal M. Elenkova M. Evensky J. Stein S. H. (2018). Periodontal disease and osteoporosis-shared risk factors and potentiation of pathogenic mechanisms. Curr. Oral Health Rep. 5, 26–32. 10.1007/s40496-018-0167-1

[B24] Bi J. Koivisto L. Dai J. Zhuang D. Jiang G. Larjava M.. (2020). Epidermal growth factor receptor signaling suppresses αvβ6 integrin and promotes periodontal inflammation and bone loss. J. Cell Sci. 133:jcs236588. 10.1242/jcs.236588 31722981

[B25] Bobetsis Y. A. Graziani F. Gürsoy M. Madianos P. N. (2020). Periodontal disease and adverse pregnancy outcomes. Periodontology 2000 83, 154–174. 10.1111/prd.12294 32385871

[B26] Bonner M. Fresno M. Gironés N. Guillén N. Santi-Rocca J. (2018). Reassessing the role of *Entamoeba gingivalis* in periodontitis. Front. Cell. Infect. Microbial. 8:379. 10.3389/fcimb.2018.00379 30420943 PMC6215854

[B27] Borish L. C. Steinke J. W. (2003). Cytokines and chemokines. J. Allergy Clin. Immunol. 111, S460–S475. 10.1067/mai.2003.108 12592293

[B28] Bostanci N. Abe T. Belibasakis G. N. Hajishengallis G. (2019). TREM-1 is upregulated in experimental periodontitis, and its blockade inhibits IL-17A and RANKL expression and suppresses bone loss. J. Clin. Med. 8:1579. 10.3390/jcm8101579 31581596 PMC6832657

[B29] Bostanci N. Bao K. (2017). Contribution of proteomics to our understanding of periodontal inflammation. Proteomics 17:1500518. 10.1002/pmic.201500518 27995754

[B30] Botelho J. Leira Y. Viana J. Machado V. Lyra P. Aldrey J. M.. (2021). The role of inflammatory diet and vitamin d on the link between periodontitis and cognitive function: a mediation analysis in older adults. Nutrients 13:924. 10.3390/nu13030924 33809193 PMC8001166

[B31] Botros N. Iyer P. Ojcius D. M. (2020). Is there an association between oral health and severity of covid-19 complications? Biomed. J. 43, 325–327. 10.1016/j.bj.2020.05.016 32713780 PMC7258848

[B32] Bui F. Q. Almeida-da Silva C. L. C. Huynh B. Trinh A. Liu J. Woodward J.. (2019). Association between periodontal pathogens and systemic disease. Biomed. J. 42, 27–35. 10.1016/j.bj.2018.12.001 30987702 PMC6468093

[B33] Bunte K. Beikler T. (2019). Th17 cells and the il-23/il-17 axis in the pathogenesis of periodontitis and immune-mediated inflammatory diseases. Int. J. Mol. Sci. 20:3394. 10.3390/ijms20143394 31295952 PMC6679067

[B34] Cardoso E. M. Reis C. Manzanares-Céspedes M. C. (2018). Chronic periodontitis, inflammatory cytokines, and interrelationship with other chronic diseases. Postgrad. Med. 130, 98–104. 10.1080/00325481.2018.1396876 29065749

[B35] Carrizales-Sepúlveda E. F. Ordaz-Farías A. Vera-Pineda R. Flores-Ramírez R. (2018). Periodontal disease, systemic inflammation and the risk of cardiovascular disease. Heart Lung Circul. 27, 1327–1334. 10.1016/j.hlc.2018.05.102 29903685

[B36] Carvalho-Filho P. C. Moura-Costa L. F. Pimentel A. Lopes M. P. Freitas S. A. Miranda P. M.. (2019). Apoptosis transcriptional profile induced by *Porphyromonas gingivalis* hmuy. Mediat. Inflamm. 2019:6758159. 10.1155/2019/6758159 31011284 PMC6442302

[B37] Ceccarelli F. Saccucci M. Di Carlo G. Lucchetti R. Pilloni A. Pranno N.. (2019). Periodontitis and rheumatoid arthritis: the same inflammatory mediators? Mediat. Inflamm. 2019:6034546. 10.1155/2019/6034546 31191116 PMC6525860

[B38] Cecoro G. Annunziata M. Iuorio M. T. Nastri L. Guida L. (2020). Periodontitis, low-grade inflammation and systemic health: a scoping review. Medicina 56:272. 10.3390/medicina56060272 32486269 PMC7353850

[B39] Chen K. Kolls J. K. (2017). Interluekin-17a (IL17A). Gene 614, 8–14. 10.1016/j.gene.2017.01.016 28122268 PMC5394985

[B40] Cheng R. Billet S. Liu C. Haldar S. Choudhury D. Tripathi M.. (2020). Periodontal inflammation recruits distant metastatic breast cancer cells by increasing myeloid-derived suppressor cells. Oncogene 39, 1543–1556. 10.1038/s41388-019-1084-z 31685946 PMC7018659

[B41] Cheng R. Feng Y. Zhang R. Liu W. Lei L. Hu T. (2018). The extent of pyroptosis varies in different stages of apical periodontitis. Biochim. Biophys. Acta 1864, 226–237. 10.1016/j.bbadis.2017.10.025 29066283

[B42] Chi A. C. Neville B. W. Krayer J. W. Gonsalves W. C. (2010). Oral manifestations of systemic disease. Am. Fam. Phys. 82, 1381–1388. 21121523

[B43] Chung M. York B. R. Michaud D. S. (2019). Oral health and cancer. Curr. Oral Health Rep. 6, 130–137. 10.1007/s40496-019-0213-7 31871854 PMC6927401

[B44] Coke C. J. Davison B. Fields N. Fletcher J. Rollings J. Roberson L.. (2021). Sars-cov-2 infection and oral health: therapeutic opportunities and challenges. J. Clin. Med. 10:156. 10.3390/jcm10010156 33466289 PMC7795434

[B45] Corbella S. Veronesi P. Galimberti V. Weinstein R. Del Fabbro M. Francetti L. (2018). Is periodontitis a risk indicator for cancer? A meta-analysis. PLoS ONE 13:e0195683. 10.1371/journal.pone.0195683 29664916 PMC5903629

[B46] Czesnikiewicz-Guzik M. Osmenda G. Siedlinski M. Nosalski R. Pelka P. Nowakowski D.. (2019). Causal association between periodontitis and hypertension: evidence from mendelian randomization and a randomized controlled trial of non-surgical periodontal therapy. Eur. Heart J. 40, 3459–3470. 10.1093/eurheartj/ehz646 31504461 PMC6837161

[B47] da Silva M. K. de Carvalho A. C. G. Alves E. H. P. da Silva F. R. P. Pessoa L. d. S. Vasconcelos D. F. P. (2017). Genetic factors and the risk of periodontitis development: findings from a systematic review composed of 13 studies of meta-analysis with 71,531 participants. Int. J. Dent. 2017:1914073. 10.1155/2017/1914073 28529526 PMC5424192

[B48] Dahlen G. Basic A. Bylund J. (2019). Importance of virulence factors for the persistence of oral bacteria in the inflamed gingival crevice and in the pathogenesis of periodontal disease. J. Clin. Med. 8:1339. 10.3390/jcm8091339 31470579 PMC6780532

[B49] D'aiuto F. Nibali L. Parkar M. Patel K. Suvan J. Donos N. (2010). Oxidative stress, systemic inflammation, and severe periodontitis. J. Dent. Res. 89, 1241–1246. 10.1177/0022034510375830 20739696 PMC3318025

[B50] Dal-Fabbro R. Marques-de Almeida M. Cosme-Silva L. Ervolino E. Cintra L. Gomes-Filho J. (2019). Chronic alcohol consumption increases inflammation and osteoclastogenesis in apical periodontitis. Int. Endodont. J. 52, 329–336. 10.1111/iej.13014 30218448

[B51] De Almeida C. V. de Camargo M. R. Russo E. Amedei A. (2019). Role of diet and gut microbiota on colorectal cancer immunomodulation. World J. Gastroenterol. 25:151. 10.3748/wjg.v25.i2.151 30670906 PMC6337022

[B52] de Molon R. S. Rossa C. Jr, Thurlings, R. M. Cirelli J. A. Koenders M. I. (2019). Linkage of periodontitis and rheumatoid arthritis: current evidence and potential biological interactions. Int. J. Mol. Sci. 20:4541. 10.3390/ijms20184541 31540277 PMC6769683

[B53] Deng F. E. Shivappa N. Tang Y. Mann J. R. Hebert J. R. (2017). Association between diet-related inflammation, all-cause, all-cancer, and cardiovascular disease mortality, with special focus on prediabetics: findings from NHANES III. Eur. J. Nutr. 56, 1085–1093. 10.1007/s00394-016-1158-4 26825592

[B54] Dessaune N. Porpino M. T. M. Antunes H. d. S. Rodrigues R. C. V. Perez A. R. Pires F. R.. (2018). Pro-inflammatory and anti-inflammatory cytokine expression in post-treatment apical periodontitis. J. Appl. Oral Sci. 26:e20170455. 10.1590/1678-7757-2017-0455 29898177 PMC5963913

[B55] Díaz C. M. Bullon B. Ruiz-Salmerón R. J. Fernández-Riejos P. Fernández-Palacín A. Battino M.. (2020). Molecular inflammation and oxidative stress are shared mechanisms involved in both myocardial infarction and periodontitis. J. Periodontal Res. 55, 519–528. 10.1111/jre.12739 32106337

[B56] Diomede F. Thangavelu S. R. Merciaro I. D'Orazio M. Bramanti P. Mazzon E. Trubiani O. (2017). *Porphyromonas gingivalis* lipopolysaccharide stimulation in human periodontal ligament stem cells: role of epigenetic modifications to the inflammation. Eur. J. Histochem. 61:2826. 10.4081/ejh.2017.2826 29046054 PMC5575416

[B57] Dizdar O. Hayran M. Guven D. C. Yılmaz T. B. Taheri S. Akman A. C.. (2017). Increased cancer risk in patients with periodontitis. Curr. Med. Res. Opin. 33, 2195–2200. 10.1080/03007995.2017.1354829 28699803

[B58] Duan Y. An W. Wu Y. Wang J. (2020). Tetramethylpyrazine reduces inflammation levels and the apoptosis of LPS-stimulated human periodontal ligament cells via the downregulation of MIR-302b. Int. J. Mol. Med. 45, 1918–1926. 10.3892/ijmm.2020.4554 32236610 PMC7169953

[B59] Duka M. Eraković M. Dolićanin Z. Stefanović D. Čolić M. (2019). Production of soluble receptor activator of nuclear factor kappa-b ligand and osteoprotegerin by apical periodontitis cells in culture and their modulation by cytokines. Mediat. Inflamm. 2019:8325380. 10.1155/2019/8325380 31011287 PMC6442274

[B60] Ebersole J. L. Graves C. L. Gonzalez O. A. Dawson D. III. Morford L. A. Huja P. E.. (2016). Aging, inflammation, immunity and periodontal disease. Periodontology 2000 72, 54–75. 10.1111/prd.12135 27501491

[B61] Elisetti N. (2021). Periodontal pocket and covid-19: could there be a possible link? Med. Hypothes. 146:110355. 10.1016/j.mehy.2020.110355 33183854 PMC7604063

[B62] Fabri G. M. C. (2020). Potential link between covid-19 and periodontitis: cytokine storm, immunosuppression, and dysbiosis. Oral Health Dent. Manage. 20, 1–5.

[B63] Ferguson B. Bokka N. R. Maddipati K. R. Ayilavarapu S. Weltman R. Zhu L.. (2020). Distinct profiles of specialized pro-resolving lipid mediators and corresponding receptor gene expression in periodontal inflammation. Front. Immunol. 11:1307. 10.3389/fimmu.2020.01307 32670289 PMC7330171

[B64] Ferlazzo N. Curro M. Isola G. Maggio S. Bertuccio M. P. Trovato-Salinaro A.. (2021). Changes in the biomarkers of oxidative/nitrosative stress and endothelial dysfunction are associated with cardiovascular risk in periodontitis patients. Curr. Issues Mol. Biol. 43, 704–715. 10.3390/cimb43020051 34287264 PMC8929118

[B65] Fine N. Chadwick J. Sun C. Parbhakar K. Khoury N. Barbour A.. (2020). Periodontal inflammation primes the systemic innate immune response. J. Dent. Res. 2020:0022034520963710. 10.1177/0022034520963710 33078669

[B66] Fitzpatrick S. G. Katz J. (2010). The association between periodontal disease and cancer: a review of the literature. J. Dent. 38, 83–95. 10.1016/j.jdent.2009.10.007 19895866

[B67] Franco C. Patricia H.-R. Timo S. Claudia B. Marcela H. (2017). Matrix metalloproteinases as regulators of periodontal inflammation. Int. J. Mol. Sci. 18:440. 10.3390/ijms18020440 28218665 PMC5343974

[B68] Fujita T. Hayashida K. Shiba H. Kishimoto A. Matsuda S. Takeda K.. (2010). The expressions of claudin-1 and e-cadherin in junctional epithelium. J. Periodont. Res. 45, 579–582. 10.1111/j.1600-0765.2009.01258.x 20337884

[B69] Fujita T. Yoshimoto T. Kajiya M. Ouhara K. Matsuda S. Takemura T.. (2018). Regulation of defensive function on gingival epithelial cells can prevent periodontal disease. Jpn. Dent. Sci. Rev. 54, 66–75. 10.1016/j.jdsr.2017.11.003 29755617 PMC5944110

[B70] Furutama D. Matsuda S. Yamawaki Y. Hatano S. Okanobu A. Memida T.. (2020). Il-6 induced by periodontal inflammation causes neuroinflammation and disrupts the blood-brain barrier. Brain Sci. 10:679. 10.3390/brainsci10100679 32992470 PMC7599694

[B71] Gallimidi A. B. Fischman S. Revach B. Bulvik R. Maliutina A. Rubinstein A. M.. (2015). Periodontal pathogens porphyromonas gingivalis and fusobacterium nucleatum promote tumor progression in an oral-specific chemical carcinogenesis model. Oncotarget 6:22613. 10.18632/oncotarget.4209 26158901 PMC4673186

[B72] Galv ao-Moreira L. V. da Cruz M. C. F. N. (2016). Oral microbiome, periodontitis and risk of head and neck cancer. Oral Oncol. 53, 17–19. 10.1016/j.oraloncology.2015.11.013 26684542

[B73] Ganesh P. Karthikeyan R. Muthukumaraswamy A. Anand J. (2017). A potential role of periodontal inflammation in Alzheimer's disease: a review. Oral Health Prev. Dent. 15, 7–12. 10.3290/j.ohpd.a37708 28232969

[B74] Garlet G. (2010). Destructive and protective roles of cytokines in periodontitis: a re-appraisal from host defense and tissue destruction viewpoints. J. Dent. Res. 89, 1349–1363. 10.1177/0022034510376402 20739705

[B75] Gil Montoya J. A. Barrios R. Sanchez-Lara I. Ramos P. Carnero C. Fornieles F.. (2020). Systemic inflammatory impact of periodontitis on cognitive impairment. Gerodontology 37, 11–18. 10.1111/ger.12431 31347730

[B76] Gomes-Filho I. S. Cruz S. S. D. Trindade S. C. Passos-Soares J. d. S. Carvalho-Filho P. C. Figueiredo A. C. M. G.. (2020). Periodontitis and respiratory diseases: a systematic review with meta-analysis. Oral Dis. 26, 439–446. 10.1111/odi.13228 31715080

[B77] Gómez-Ba nuelos E. Mukherjee A. Darrah E. Andrade F. (2019). Rheumatoid arthritis-associated mechanisms of *Porphyromonas gingivalis* and *Aggregatibacter actinomycetemcomitans. J. Clin. Med*. 8:1309. 10.3390/jcm8091309 31454946 PMC6780899

[B78] González-Febles J. Sanz M. (2021). Periodontitis and rheumatoid arthritis: what have we learned about their connection and their treatment? Periodontology 2000 87, 181–203. 10.1111/prd.12385 34463976

[B79] Górski B. Nargiełło E. Opolski G. Ganowicz E. Gorska R. (2016). The association between dental status and systemic lipid profile and inflammatory mediators in patients after myocardial infarction. Adv. Clin. Exp. Med. 25, 625–630. 10.17219/acem/62937 27629835

[B80] Graves D. (2008). Cytokines that promote periodontal tissue destruction. J. Periodontal. 79, 1585–1591. 10.1902/jop.2008.080183 18673014

[B81] Grønkjær L. L. Holmstrup P. Schou S. Kongstad J. Jepsen P. Vilstrup H. (2018). Periodontitis in patients with cirrhosis: a cross-sectional study. BMC Oral Health 18:22. 10.1186/s12903-018-0487-5 29439734 PMC5811961

[B82] Güler B. Doǧan E. Onbaşi K. (2020). The relationship between monocyte count to high-density lipoprotein ratio and severity of inflammation in aggressive periodontitis: a retrospective analysis. Meandros Med. Dent. J. 21:122. 10.4274/meandros.galenos.2020.41033

[B83] Gupta S. Sahni V. (2020). The intriguing commonality of netosis between covid-19; periodontal disease. Med. Hypothes. 144:109968. 10.1016/j.mehy.2020.109968 32534340 PMC7276117

[B84] Gusman D. J. R. Ervolino E. Theodoro L. H. Garcia V. G. Nagata M. J. H. Alves B. E. S.. (2019). Antineoplastic agents exacerbate periodontal inflammation and aggravate experimental periodontitis. J. Clin. Periodontal. 46, 457–469. 10.1111/jcpe.13101 30854670

[B85] Ha N. H. Woo B. H. Kim D. J. Ha E. S. Choi J. I. Kim S. J.. (2015). Prolonged and repetitive exposure to *Porphyromonas gingivalis* increases aggressiveness of oral cancer cells by promoting acquisition of cancer stem cell properties. Tumor Biol. 36, 9947–9960. 10.1007/s13277-015-3764-9 26178482

[B86] Hajishengallis G. (2015). Periodontitis: from microbial immune subversion to systemic inflammation. Nat. Rev. Immunol. 15, 30–44. 10.1038/nri3785 25534621 PMC4276050

[B87] Hajishengallis G. (2020). New developments in neutrophil biology and periodontitis. Periodontology 2000 82, 78–92. 10.1111/prd.12313 31850633

[B88] Hajishengallis G. Kajikawa T. Hajishengallis E. Maekawa T. Li X. Belibasakis G. N.. (2020). “Complement C3 as a target of host modulation in periodontitis,” in Emerging Therapies in Periodontics, ed S. Sahingur (Springer), 13–29. 10.1007/978-3-030-42990-4_2

[B89] Hajishengallis G. Moutsopoulos N. M. (2016). Role of bacteria in leukocyte adhesion deficiency-associated periodontitis. Microb. Pathog. 94, 21–26. 10.1016/j.micpath.2015.09.003 26375893 PMC4791199

[B90] Hamilton J. A. Hasturk H. Kantarci A. Serhan C. N. Van Dyke T. (2017). Atherosclerosis, periodontal disease, and treatment with resolvins. Curr. Atheroscl. Rep. 19:57. 10.1007/s11883-017-0696-4 29110146

[B91] Han Y. Wang X. (2013). Mobile microbiome: oral bacteria in extra-oral infections and inflammation. J. Dent. Res. 92, 485–491. 10.1177/0022034513487559 23625375 PMC3654760

[B92] Hashioka S. Inoue K. Hayashida M. Wake R. Oh-Nishi A. Miyaoka T. (2018). Implications of systemic inflammation and periodontitis for major depression. Front. Neurosci. 12:483. 10.3389/fnins.2018.00483 30072865 PMC6058051

[B93] Hasturk H. Kantarci A. (2015). Activation and resolution of periodontal inflammation and its systemic impact. Periodontology 2000 69, 255–273. 10.1111/prd.12105 26252412 PMC4530469

[B94] Haworth S. Kho P. F. Holgerson P. L. Hwang L.-D. Timpson N. J. Rentería M. E.. (2021). Assessment and visualization of phenome-wide causal relationships using genetic data: an application to dental caries and periodontitis. Eur. J. Hum. Genet. 29, 300–308. 10.1038/s41431-020-00734-4 33011735 PMC7868372

[B95] Heikkilä P. But A. Sorsa T. Haukka J. (2018). Periodontitis and cancer mortality: register-based cohort study of 68,273 adults in 10-year follow-up. Int. J. Cancer 142, 2244–2253. 10.1002/ijc.31254 29322513

[B96] Hernández-Monjaraz B. Santiago-Osorio E. Monroy-García A. Ledesma-Martínez E. Mendoza-Nú nez V. M. (2018). Mesenchymal stem cells of dental origin for inducing tissue regeneration in periodontitis: a mini-review. Int. J. Mol. Sci. 19:944. 10.3390/ijms19040944 29565801 PMC5979585

[B97] Hickey N. A. Shalamanova L. Whitehead K. A. Dempsey-Hibbert N. van der Gast C. Taylor R. L. (2020). Exploring the putative interactions between chronic kidney disease and chronic periodontitis. Crit. Rev. Microbiol. 46, 61–77. 10.1080/1040841X.2020.1724872 32046541

[B98] Hirschfeld J. Higham J. Chatzistavrianou D. Blair F. Richards A. Chapple I. L. (2019). Systemic disease or periodontal disease? Distinguishing causes of gingival inflammation: a guide for dental practitioners. Part 1: immune-mediated, autoinflammatory, and hereditary lesions. Br. Dent. J. 227, 961–966. 10.1038/s41415-019-1050-8 31844223

[B99] Hoare A. Soto C. Rojas-Celis V. Bravo D. (2019). Chronic inflammation as a link between periodontitis and carcinogenesis. Mediat. Inflamm. 2019:1029857. 10.1155/2019/1029857 31049022 PMC6458883

[B100] Holmström S. B. Clark R. Zwicker S. Bureik D. Kvedaraite E. Bernasconi E.. (2017). Gingival tissue inflammation promotes increased matrix metalloproteinase-12 production by cd200rlow monocyte-derived cells in periodontitis. J. Immunol. 199, 4023–4035. 10.4049/jimmunol.1700672 29101312

[B101] Holmström S. B. Lira-Junior R. Zwicker S. Majster M. Gustafsson A. Åkerman S.. (2019). Mmp-12 and s100s in saliva reflect different aspects of periodontal inflammation. Cytokine 113, 155–161. 10.1016/j.cyto.2018.06.036 29983358

[B102] Hong J.-Y. Lee J.-S. Choi S.-H. Shin H.-S. Park J.-C. Shin S.-I.. (2019). A randomized, double-blind, placebo-controlled multicenter study for evaluating the effects of fixed-dose combinations of vitamin c, vitamin e, lysozyme, and carbazochrome on gingival inflammation in chronic periodontitis patients. BMC Oral Health 19, 1–8. 10.1186/s12903-019-0728-2 30845920 PMC6407240

[B103] Hu B. Huang S. Yin L. (2021). The cytokine storm and covid-19. J. Med. Virol. 93, 250–256. 10.1002/jmv.26232 32592501 PMC7361342

[B104] Huang J. Cai X. Ou Y. Fan L. Zhou Y. Wang Y. (2019). Protective roles of ficz and aryl hydrocarbon receptor axis on alveolar bone loss and inflammation in experimental periodontitis. J. Clin. Periodontal. 46, 882–893. 10.1111/jcpe.13166 31286538

[B105] Hujoel P. P. Drangsholt M. Spiekerman C. Weiss N. S. (2003). An exploration of the periodontitis-cancer association. Ann. Epidemiol. 13, 312–316. 10.1016/S1047-2797(02)00425-8 12821269

[B106] Iddir M. Brito A. Dingeo G. Fernandez Del Campo S. S. Samouda H. La Frano M. R.. (2020). Strengthening the immune system and reducing inflammation and oxidative stress through diet and nutrition: considerations during the covid-19 crisis. Nutrients 12:1562. 10.3390/nu12061562 32471251 PMC7352291

[B107] Ide M. (2021). “Periodontal disease and systemic health,” in Periodontology, ed R. Palmer, and P. Floyd (Springer), 31–43. 10.1007/978-3-030-76243-8_3

[B108] Im S. I. Heo J. Kim B. J. Cho K.-I. Kim H. S. Heo J. H.. (2018). Impact of periodontitis as representative of chronic inflammation on long-term clinical outcomes in patients with atrial fibrillation. Open Heart 5:e000708. 10.1136/openhrt-2017-000708 29713482 PMC5922561

[B109] Irani S. Barati I. Badiei M. (2020). Periodontitis and oral cancer-current concepts of the etiopathogenesis. Oncol. Rev. 14:465. 10.4081/oncol.2020.465 32231765 PMC7097927

[B110] Iranmanesh B. Khalili M. Amiri R. Zartab H. Aflatoonian M. (2021). Oral manifestations of covid-19 disease: a review article. Dermatol. Ther. 34:e14578. 10.1111/dth.14578 33236823 PMC7744903

[B111] Jafaripour S. Sedighi S. Jokar M. H. Aghaei M. Moradzadeh M. (2020). Inflammation, diet, and type 2 diabetes: a mini-review. J. Immun. Immunochem. 41, 768–777. 10.1080/15321819.2020.1750423 32397924

[B112] Jepsen S. Suvan J. Deschner J. (2020). The association of periodontal diseases with metabolic syndrome and obesity. Periodontology 2000 83, 125–153. 10.1111/prd.12326 32385882

[B113] Jia L. Han N. Du J. Guo L. Luo Z. Liu Y. (2019). Pathogenesis of important virulence factors of *Porphyromonas gingivalis* via toll-like receptors. Front. Cell. Infect. microbiol. 9:262. 10.3389/fcimb.2019.00262 31380305 PMC6657652

[B114] Jin S.-H. Zhou R.-H. Guan X.-Y. Zhou J.-G. Liu J.-G. (2020). Identification of novel key lncrnas involved in periodontitis by weighted gene co-expression network analysis. J. Periodontal Res. 55, 96–106. 10.1111/jre.12693 31512745

[B115] Jockel-Schneider Y. Schlagenhauf U. Stölzel P. Goßner S. Carle R. Ehmke B.. (2021). Nitrate-rich diet alters the composition of the oral microbiota in periodontal recall patients. J. Periodontol. 1–10. 10.1002/JPER.20-0778 33742692

[B116] Jurdziński K. T. Potempa J. Grabiec A. M. (2020). Epigenetic regulation of inflammation in periodontitis: cellular mechanisms and therapeutic potential. Clin. Epigenet. 12, 1–18. 10.1186/s13148-020-00982-7 33256844 PMC7706209

[B117] Kato R. Ishihara Y. Kawanabe N. Sumiyoshi K. Yoshikawa Y. Nakamura M.. (2013). Gap-junction-mediated communication in human periodontal ligament cells. J. Dent. Res. 92, 635–640. 10.1177/0022034513489992 23677649

[B118] Katsifis G. E. Rekka S. Moutsopoulos N. M. Pillemer S. Wahl S. M. (2009). Systemic and local interleukin-17 and linked cytokines associated with sjögren's syndrome immunopathogenesis. Am. J. Pathol. 175, 1167–1177. 10.2353/ajpath.2009.090319 19700754 PMC2731135

[B119] Kaur T. Uppoor A. Naik D. (2016). Parkinson's disease and periodontitis-the missing link? A review. Gerodontology 33, 434–438. 10.1111/ger.12188 25664991

[B120] Kavrikova D. Borilova Linhartova P. Lucanova S. Poskerova H. Fassmann A. Izakovicova Holla L. (2019). Chemokine receptor 2 (CXCR2) gene variants and their association with periodontal bacteria in patients with chronic periodontitis. Mediat. Inflamm. 2019:2061868. 10.1155/2019/2061868 30863202 PMC6378799

[B121] Khumaedi A. I. Purnamasari D. Wijaya I. P. Soeroso Y. (2019). The relationship of diabetes, periodontitis and cardiovascular disease. Diabetes Metabol. Syndr. Clin. Res. Rev. 13, 1675–1678. 10.1016/j.dsx.2019.03.023 31336540

[B122] Kim D. Lee G. Huh Y. Lee S. Park K. Kim S. Kim J. Koh J. Ryu J. (2017). Nampt is an essential regulator of RA-mediated periodontal inflammation. J. Dent. Res. 96, 703–711. 10.1177/0022034517690389 28165872

[B123] Kim H. Kim M.-B. Kim C. Hwang J.-K. (2018). Inhibitory effects of panduratin a on periodontitis-induced inflammation and osteoclastogenesis through inhibition of MAPK pathways *in vitro*. *J. Microbiol. Biotechnol*. 28, 190–198. 10.4014/jmb.1707.07042 29061028

[B124] Kinane D. F. Stathopoulou P. G. Papapanou P. N. (2017). Periodontal diseases. Nat. Rev. Dis. Primers 3, 1–14. 10.1038/nrdp.2017.38 28805207

[B125] Kırzıoğlu F. Y. Bulut M. T. Doğan B. Fentoğlu Ö. Özmen Ö. Çarsancaklı S. A.. (2017). Anti-inflammatory effect of rosuvastatin decreases alveolar bone loss in experimental periodontitis. J. Oral Sci. 59, 247–255. 10.2334/josnusd.16-0398 28637984

[B126] Konkel J. E. O'Boyle C. Krishnan S. (2019). Distal consequences of oral inflammation. Front. Immunol. 10:1403. 10.3389/fimmu.2019.01403 31293577 PMC6603141

[B127] Könönen E. Gursoy M. Gursoy U. K. (2019). Periodontitis: a multifaceted disease of tooth-supporting tissues. J. Clin. Med. 8:1135. 10.3390/jcm8081135 31370168 PMC6723779

[B128] Koopman M. El Aidy S. (2017). Depressed gut? The microbiota-diet-inflammation trialogue in depression. Curr. Opin. Psychiatry 30, 369–377. 10.1097/YCO.0000000000000350 28654462

[B129] Kotsakis G. A. Chrepa V. Shivappa N. Wirth M. Hébert J. Koyanagi A.. (2018). Diet-borne systemic inflammation is associated with prevalent tooth loss. Clin. Nutr. 37, 1306–1312. 10.1016/j.clnu.2017.06.001 28633943 PMC5723246

[B130] Kovell L. C. Yeung E. H. Miller E. R. III. Appel L. J. Christenson R. H. Rebuck H.. (2020). Healthy diet reduces markers of cardiac injury and inflammation regardless of macronutrients: results from the omniheart trial. Int. J. Cardiol. 299, 282–288. 10.1016/j.ijcard.2019.07.102 31447226 PMC7172033

[B131] Küchler E. C. Hannegraf N. D. Lara R. M. Reis C. L. B. de Oliveira D. S. B. Mazzi-Chaves J. F.. (2021). Investigation of genetic polymorphisms in BMP2, BMP4, SMAD6, and RUNX2 and persistent apical periodontitis. J. Endodont. 47, 278–285. 10.1016/j.joen.2020.11.014 33245975

[B132] Kumar P. S. (2017). From focal sepsis to periodontal medicine: a century of exploring the role of the oral microbiome in systemic disease. J. Physiol. 595, 465–476. 10.1113/JP272427 27426277 PMC5233655

[B133] Kurgan S. Kantarci A. (2018). Molecular basis for immunohistochemical and inflammatory changes during progression of gingivitis to periodontitis. Periodontology 2000 76, 51–67. 10.1111/prd.12146 29194785

[B134] Laine M. L. Crielaard W. Loos B. G. (2012). Genetic susceptibility to periodontitis. Periodontology 2000 58, 37–68. 10.1111/j.1600-0757.2011.00415.x 22133366

[B135] Lamster I. B. Pagan M. (2017). Periodontal disease and the metabolic syndrome. Int. Dent. J. 67, 67–77. 10.1111/idj.12264 27861820 PMC9376683

[B136] Leblhuber F. Huemer J. Steiner K. Gostner J. M. Fuchs D. (2020). Knock-on effect of periodontitis to the pathogenesis of Alzheimer's disease? Wiener Kiln. Wochensch. 132, 1–6. 10.1007/s00508-020-01638-5 32215721 PMC7519001

[B137] Lee C.-T. Teles R. Kantarci A. Chen T. McCafferty J. Starr J. R.. (2016). Resolvin E1 reverses experimental periodontitis and dysbiosis. J. Immunol. 197, 2796–2806. 10.4049/jimmunol.1600859 27543615 PMC5026932

[B138] Leira Y. Ameijeira P. Domínguez C. López-Arias E. Ávila-Gómez P. Pérez-Mato M.. (2019a). Periodontal inflammation is related to increased serum calcitonin gene-related peptide levels in patients with chronic migraine. J. Periodontal. 90, 1088–1095. 10.1002/JPER.19-0051 31070784

[B139] Leira Y. Carballo Á. Orlandi M. Aldrey J. M. Pías-Peleteiro J. M. Moreno F.. (2020a). Periodontitis and systemic markers of neurodegeneration: a case-control study. J. Clin. Periodontol. 47, 561–571. 10.1111/jcpe.13267 32027386

[B140] Leira Y. Orlandi M. Czesnikiewicz-Guzik M. Guzik T. Hingorani A. Nart J. D'Aiuto F.. (2020b). Is systemic inflammation a missing link between periodontitis and hypertension? Results from two large populations-based surveys. J. Intern. Med. 289, 532–546. 10.1111/joim.13180 32969093

[B141] Leira Y. Rodríguez-Yá nez M. Arias S. López-Dequidt I. Campos F. Sobrino T.. (2019b). Periodontitis is associated with systemic inflammation and vascular endothelial dysfunction in patients with lacunar infarct. J. Periodontal. 90, 465–474. 10.1002/JPER.18-0560 30417380

[B142] Li J. Lu H. Wu H. Huang S. Chen L. Gui Q.. (2020). Periodontitis in elderly patients with type 2 diabetes mellitus: impact on gut microbiota and systemic inflammation. Aging 12, 25956–25980. 10.18632/aging.202174 33234730 PMC7803515

[B143] Li J. Xiao X. Wei W. Ding H. Yue Y. Tian Y.. (2019a). Inhibition of angiotensin II receptor I prevents inflammation and bone loss in periodontitis. J. Periodontal. 90, 208–216. 10.1002/JPER.17-0753 30066953

[B144] Li L. Bao J. Chen B. Yan F. (2021). High-fat diet promotes the impact of periodontitis on gut microbiota and glucose metabolism. Chinese J. Stomatol. 56, 539–548. 10.3760/cma.j.cn112144-20210123-00037 34098669

[B145] Li X. Hu L. Ma L. Chang S. Wang W. Feng Y.. (2019b). Severe periodontitis may influence cementum and dental pulp through inflammation, oxidative stress, and apoptosis. J. Periodontol. 90, 1297–1306. 10.1002/JPER.18-0604 31161648

[B146] Lira-Junior R. Figueredo C. M. (2016). Periodontal and inflammatory bowel diseases: is there evidence of complex pathogenic interactions? World J. Gastroenterol. 22:7963. 10.3748/wjg.v22.i35.7963 27672291 PMC5028810

[B147] Listyarifah D. Al-Samadi A. Salem A. Syaify A. Salo T. Tervahartiala T.. (2017). Infection and apoptosis associated with inflammation in periodontitis: an immunohistologic study. Oral Dis. 23, 1144–1154. 10.1111/odi.12711 28686335

[B148] Liu W. Cui Y. Wei J. Sun J. Zheng L. Xie J. (2020). Gap junction-mediated cell-to-cell communication in oral development and oral diseases: a concise review of research progress. Int. J. Oral Sci. 12, 1–9. 10.1038/s41368-020-0086-6 32532966 PMC7293327

[B149] Loos B. G. (2005). Systemic markers of inflammation in periodontitis. J. Periodontal. 76, 2106–2115. 10.1902/jop.2005.76.11-S.2106 16277583

[B150] Loos B. G. Van Dyke T. E. (2020). The role of inflammation and genetics in periodontal disease. Periodontology 2000 83, 26–39. 10.1111/prd.12297 32385877 PMC7319430

[B151] Lorenz K. Keller T. Noack B. Freitag A. Netuschil L. Hoffmann T. (2017). Evaluation of a novel point-of-care test for active matrix metalloproteinase-8: agreement between qualitative and quantitative measurements and relation to periodontal inflammation. J. Periodontal Res. 52, 277–284. 10.1111/jre.12392 27214099

[B152] Lourenςo T. G. B. Spencer S. J. Alm E. J. Colombo A. P. V. (2018). Defining the gut microbiota in individuals with periodontal diseases: an exploratory study. J. Oral Microbiol. 10:1487741. 10.1080/20002297.2018.1487741 29988721 PMC6032013

[B153] Maisonneuve P. Amar S. Lowenfels A. B. (2017). Periodontal disease, edentulism, and pancreatic cancer: a meta-analysis. Ann. Oncol. 28, 985–995. 10.1093/annonc/mdx019 28453689

[B154] Maldonado A. Pirracchio L. Imber J.-C. Bürgin W. Möller B. Sculean A.. (2020). Citrullination in periodontium is associated with *Porphyromonas gingivalis. Arch. Oral Biol*. 114:104695. 10.1016/j.archoralbio.2020.104695 32315811

[B155] Marouf N. Cai W. Said K. N. Daas H. Diab H. Chinta V. R.. (2021). Association between periodontitis and severity of covid-19 infection: a case-control study. J. Clin. Periodontol. 48, 483–491. 10.1111/jcpe.13435 33527378 PMC8014679

[B156] Masumoto R. Kitagaki J. Fujihara C. Matsumoto M. Miyauchi S. Asano Y.. (2019). Identification of genetic risk factors of aggressive periodontitis using genomewide association studies in association with those of chronic periodontitis. J. Periodontal Res. 54, 199–206. 10.1111/jre.12620 30303256

[B157] Mau L.-P. Kuan Y.-C. Tsai Y.-W. Lin J.-J. Huynh-Ba G. Weng P.-W.. (2017). Patients with chronic periodontitis present increased risk for osteoporosis: a population-based cohort study in Taiwan. J. Periodontal Res. 52, 922–929. 10.1111/jre.12464 28464230

[B158] McGrattan A. M. McGuinness B. McKinley M. C. Kee F. Passmore P. Woodside J. V.. (2019). Diet and inflammation in cognitive ageing and Alzheimer's disease. Curr. Nutr. Rep. 8, 53–65. 10.1007/s13668-019-0271-4 30949921 PMC6486891

[B159] Meisel P. Pink C. Pitchika V. Nauck M. Völzke H. Kocher T. (2020). Competing interplay between systemic and periodontal inflammation: obesity overrides the impact of oral periphery. Clin. Oral Invest. 25, 1–9. 10.1007/s00784-020-03514-y 32827080 PMC8238770

[B160] Mezzavilla M. Navarra C. O. Di Lenarda R. Gasparini P. Bevilacqua L. Robino A. (2021). Runs of homozygosity are associated with staging of periodontitis in isolated populations. Hum. Mol. Genet. 30, 1154–1159. 10.1093/hmg/ddab085 33772543

[B161] Michalowicz B. S. Diehl S. R. Gunsolley J. C. Sparks B. S. Brooks C. N. Koertge T. E.. (2000). Evidence of a substantial genetic basis for risk of adult periodontitis. J. Periodontal. 71, 1699–1707. 10.1902/jop.2000.71.11.1699 11128917

[B162] Michaud D. S. Fu Z. Shi J. Chung M. (2017). Periodontal disease, tooth loss, and cancer risk. Epidemiol. Rev. 39, 49–58. 10.1093/epirev/mxx006 28449041 PMC5868279

[B163] Michaud D. S. Lu J. Peacock-Villada A. Y. Barber J. R. Joshu C. E. Prizment A. E.. (2018). Periodontal disease assessed using clinical dental measurements and cancer risk in the ARIC study. J. Natl. Cancer Instit. 110, 843–854. 10.1093/jnci/djx278 29342298 PMC6093423

[B164] Minty M. Canceil T. Serino M. Burcelin R. Tercé F. Blasco-Baque V. (2019). Oral microbiota-induced periodontitis: a new risk factor of metabolic diseases. Rev. Endocr. Metab. Disord. 20, 449–459. 10.1007/s11154-019-09526-8 31741266

[B165] Miossec P. Kolls J. K. (2012). Targeting IL-17 and TH 17 cells in chronic inflammation. Nat. Rev. Drug Discov. 11, 763–776. 10.1038/nrd3794 23023676

[B166] Moghadam S. A. Shirazaiy M. Risbaf S. (2017). The associations between periodontitis and respiratory disease. J. Nepal Health Res. Council 15, 1–6. 10.3126/jnhrc.v15i1.18023 28714484

[B167] Mohr S. Amylidi-Mohr S. K. Stadelmann P. Sculean A. Persson R. Eick S. Surbek D. V. (2019). Systemic inflammation in pregnant women with periodontitis and preterm prelabor rupture of membranes: a prospective case-control study. Front. Immunol. 10:2624. 10.3389/fimmu.2019.02624 31787985 PMC6854050

[B168] Moutsopoulos N. M. Madianos P. N. (2006). Low-grade inflammation in chronic infectious diseases: paradigm of periodontal infections. Ann. N.Y. Acad. Sci. 1088, 251–264. 10.1196/annals.1366.032 17192571

[B169] Nascimento G. G. Leite F. R. Scheutz F. Lopez R. (2017). Periodontitis: from infection to inflammation. Curr. Oral Health Rep. 4, 301–308. 10.1007/s40496-017-0158-7

[B170] Nazir M. A. (2017). Prevalence of periodontal disease, its association with systemic diseases and prevention. Int. J. Health Sci. 11:72. 28539867 PMC5426403

[B171] Noack M. Miossec P. (2014). Th17 and regulatory t cell balance in autoimmune and inflammatory diseases. Autoimmun. Rev. 13, 668–677. 10.1016/j.autrev.2013.12.004 24418308

[B172] Offenbacher S. Barros S. P. Beck J. D. (2008). Rethinking periodontal inflammation. J. Periodontol. 79, 1577–1584. 10.1902/jop.2008.080220 18673013

[B173] Palioto D. B. Finoti L. S. Kinane D. F. Benakanakere M. (2019). Epigenetic and inflammatory events in experimental periodontitis following systemic microbial challenge. J. Clin. Periodontol. 46, 819–829. 10.1111/jcpe.13151 31131910 PMC6641985

[B174] Pan W. Wang Q. Chen Q. (2019). The cytokine network involved in the host immune response to periodontitis. Int. J. Oral Sci. 11, 1–13. 10.1038/s41368-019-0064-z 31685798 PMC6828663

[B175] Patnaik K. Pradeep A. Nagpal K. Karvekar S. Singh P. Raju A. (2017). Human chemerin correlation in gingival crevicular fluid and tear fluid as markers of inflammation in chronic periodontitis and type-2 diabetes mellitus. J. Invest. Clin. Dent. 8:e12181. 10.1111/jicd.12181 26224661

[B176] Paula-Silva F. W. G. Arnez M. F. M. Petean I. B. F. Almeida-Junior L. A. da Silva R. A. B. da Silva L. A. B.. (2020). Effects of 5-lipoxygenase gene disruption on inflammation, osteoclastogenesis and bone resorption in polymicrobial apical periodontitis. Arch. Oral Biol. 112:104670. 10.1016/j.archoralbio.2020.104670 32058859

[B177] Peddis N. Musu D. Ideo F. Rossi-Fedele G. Cotti E. (2019). Interaction of biologic therapy with apical periodontitis and periodontitis: a systematic review. Austr. Dent. J. 64, 122–134. 10.1111/adj.12684 30811605

[B178] Penoni D. Vettore M. Torres S. Farias M. Le ao A. (2019). An investigation of the bidirectional link between osteoporosis and periodontitis. Arch. Osteop. 14, 1–10. 10.1007/s11657-019-0643-9 31444638

[B179] Pessoa L. Aleti G. Choudhury S. Nguyen D. Yaskell T. Zhang Y.. (2019). Host-microbial interactions in systemic lupus erythematosus and periodontitis. Front. Immunol. 10:2602. 10.3389/fimmu.2019.02602 31781106 PMC6861327

[B180] Petit C. Batool F. Bugueno I. M. Schwinté P. Benkirane-Jessel N. Huck O. (2019). Contribution of statins towards periodontal treatment: a review. Mediat. Inflamm. 2019:6367402. 10.1155/2019/6367402 30936777 PMC6415285

[B181] Pietiäinen M. Liljestrand J. M. Akhi R. Buhlin K. Johansson A. Paju S.. (2019). Saliva and serum immune responses in apical periodontitis. J. Clin. Med. 8:889. 10.3390/jcm8060889 31234349 PMC6617293

[B182] Pietropaoli D. Del Pinto R. Ferri C. Marzo G. Giannoni M. Ortu E.. (2020). Association between periodontal inflammation and hypertension using periodontal inflamed surface area and bleeding on probing. J. Clin. Periodontol. 47, 160–172. 10.1111/jcpe.13216 31680283

[B183] Piras V. Usai P. Mezzena S. Susnik M. Ideo F. Schirru E. Cotti E. (2017). Prevalence of apical periodontitis in patients with inflammatory bowel diseases: a retrospective clinical study. J. Endodont. 43, 389–394. 10.1016/j.joen.2016.11.004 28231978

[B184] Poole S. Singhrao S. K. Chukkapalli S. Rivera M. Velsko I. Kesavalu L.. (2015). Active invasion of *Porphyromonas gingivalis* and infection-induced complement activation in apoe-/-mice brains. J. Alzheimer's Dis. 43, 67–80. 10.3233/JAD-140315 25061055

[B185] Poole S. Singhrao S. K. Kesavalu L. Curtis M. A. Crean S. (2013). Determining the presence of periodontopathic virulence factors in short-term postmortem Alzheimer's disease brain tissue. J. Alzheimer's Dis. 36, 665–677. 10.3233/JAD-121918 23666172

[B186] Prana V. Tieri P. Palumbo M. C. Mancini E. Castiglione F. (2019). Modeling the effect of high calorie diet on the interplay between adipose tissue, inflammation, and diabetes. Comput. Math. Methods Med. 2019:7525834. 10.1155/2019/7525834 30863457 PMC6378014

[B187] Preshaw P. M. Taylor J. J. Jaedicke K. M. De Jager M. Bikker J. W. Selten W.. (2020). Treatment of periodontitis reduces systemic inflammation in type 2 diabetes. J. Clin. Periodontol. 47, 737–746. 10.1111/jcpe.13274 32106333

[B188] Purnamasari D. Khumaedi A. I. Soeroso Y. Marhamah S. (2019). The influence of diabetes and or periodontitis on inflammation and adiponectin level. Diab. Metab. Syndrome 13, 2176–2182. 10.1016/j.dsx.2019.05.012 31235154

[B189] Ramadan D. E. Hariyani N. Indrawati R. Ridwan R. D. Diyatri I. (2020). Cytokines and chemokines in periodontitis. Eur. J. Dent. 14:483. 10.1055/s-0040-1712718 32575137 PMC7440949

[B190] Razquin C. Martinez-Gonzalez M. A. (2019). A traditional mediterranean diet effectively reduces inflammation and improves cardiovascular health. Nutrients 11:1842. 10.3390/nu11081842 31395816 PMC6723673

[B191] Reis A. A. Paz H. E. d. S. Monteiro M. d. F. Casati M. Z. Steiner-Oliveira C. Pascon F. M.. (2021). Early manifestation of periodontal disease in children and its association with familial aggregation. J. Dentist. Child 88, 140–143. 34321147

[B192] Rowińska I. Szyperska-Ślaska A. Zariczny P. Pasławski R. Kramkowski K. Kowalczyk P. (2021). The influence of diet on oxidative stress and inflammation induced by bacterial biofilms in the human oral cavity. Materials 14:1444. 10.3390/ma14061444 33809616 PMC8001659

[B193] Rudick C. P. Miyamoto T. Lang M. S. Agrawal D. K. (2017). Triggering receptor expressed on myeloid cells in the pathogenesis of periodontitis: potential novel treatment strategies. Expert Rev. Clin. Immunol. 13, 1189–1197. 10.1080/1744666X.2017.1392855 29027827 PMC6064610

[B194] Sahni V. Gupta S. (2020). Covid-19 & periodontitis: the cytokine connection. Medical Hypothes. 144:109908. 10.1016/j.mehy.2020.109908 32534336 PMC7832148

[B195] Sansores-Espa na D. Carrillo-Avila A. Melgar-Rodriguez S. Díaz-Zu niga J. Martínez-Aguilar V. (2020). Periodontitis and Alzheimers disease. Med. Oral Patol. Oral Cir. Bucal. 23:23940. 10.4317/medoral.23940 32701930 PMC7806353

[B196] Saranyan R. Manovijay B. Priya K. Jayachandran D. Babu B. Raj C. S. (2017). Impact of chronic periodontitis on systemic conditions: a review. Asian J. Med. Health 1–10. 10.9734/AJMAH/2017/3851123066303

[B197] Sarda T. Rathod S. Kolte A. Bodhare G. Modak A. (2016). Expression of periodontal inflammation into left ventricular hypertrophy in type 2 diabetes mellitus: a cross-sectional study. Contemp. Clin. Dent. 7:343. 10.4103/0976-237X.188559 27630499 PMC5004548

[B198] Scannapieco F. A. (2004). Periodontal inflammation: from gingivitis to systemic disease? *Compend. Contin. Educ. Dent*. 25(7 Suppl. 1), 16–25. 15645883

[B199] Schaefer A. S. (2018). Genetics of periodontitis: discovery, biology, and clinical impact. Periodontology 2000 78, 162–173. 10.1111/prd.12232 30198130

[B200] Schenkein H. A. Papapanou P. N. Genco R. Sanz M. (2020). Mechanisms underlying the association between periodontitis and atherosclerotic disease. Periodontology 2000 83, 90–106. 10.1111/prd.12304 32385879

[B201] Schulz S. Pütz N. Jurianz E. Schaller H.-G. Reichert S. (2019). Are there any common genetic risk markers for rheumatoid arthritis and periodontal diseases? A case-control study. Mediat. Inflamm. 2019:2907062. 10.1155/2019/2907062 30890897 PMC6390239

[B202] Seymour G. Ford P. Cullinan M. Leishman S. Yamazaki K. (2007). Relationship between periodontal infections and systemic disease. Clin. Microbiol. Infect. 13, 3–10. 10.1111/j.1469-0691.2007.01798.x 17716290

[B203] Shaddox L. Mullersman A. Huang H. Wallet S. Langaee T. Aukhil I. (2017). Epigenetic regulation of inflammation in localized aggressive periodontitis. Clin. Epigenet. 9:94. 10.1186/s13148-017-0385-8 28883894 PMC5581417

[B204] Shaddox L. M. Morford L. A. Nibali L. (2021). Periodontal health and disease: the contribution of genetics. Periodontology 2000 85, 161–181. 10.1111/prd.12357 33226705 PMC13404626

[B205] Shao J. Wu L. Leng W.-D. Fang C. Zhu Y.-J. Jin Y.-H.. (2018). Periodontal disease and breast cancer: a meta-analysis of 1, 73,162 participants. Front. Oncol. 8:601. 10.3389/fonc.2018.00601 30619743 PMC6299876

[B206] Sharma N. Sharma R. K. Tewari S. Chauhan M. Bhatia A. (2018). Association of periodontal inflammation, systemic inflammation, and duration of menopausal years in postmenopausal women. Quintess. Int. 49, 123–131. 29234743 10.3290/j.qi.a39512

[B207] Shin Y. Choung H. Lee J. Rhyu I. Kim H. (2019). Association of periodontitis with oral cancer: a case-control study. J. Dent. Res. 98, 526–533. 10.1177/0022034519827565 30779879

[B208] Shungin D. Haworth S. Divaris K. Agler C. S. Kamatani Y. Lee M. K.. (2019). Genome-wide analysis of dental caries and periodontitis combining clinical and self-reported data. Nat. Commun. 10, 1–13. 10.1038/s41467-019-10630-1 31235808 PMC6591304

[B209] Silva L. M. Brenchley L. Moutsopoulos N. M. (2019). Primary immunodeficiencies reveal the essential role of tissue neutrophils in periodontitis. Immunol. Rev. 287, 226–235. 10.1111/imr.12724 30565245 PMC7015146

[B210] Sima C. Van Dyke E. T. (2016). Therapeutic targets for management of periodontitis and diabetes. Curr. Pharm. Des. 22, 2216–2237. 10.2174/1381612822666160216150338 26881443 PMC4854768

[B211] Sima C. Viniegra A. Glogauer M. (2019). Macrophage immunomodulation in chronic osteolytic diseases-the case of periodontitis. J. Leukocyte Biol. 105, 473–487. 10.1002/JLB.1RU0818-310R 30452781 PMC6386606

[B212] Singhal S. Pradeep A. R. Kanoriya D. Garg V. (2016). Human soluble receptor for advanced glycation end products and tumor necrosis factor-α as gingival crevicular fluid and serum markers of inflammation in chronic periodontitis and type 2 diabetes. J. Oral Sci. 58, 547–553. 10.2334/josnusd.16-0017 28025439

[B213] Song B. Zhang Y. Chen L. Zhou T. Huang W. Zhou X.. (2017). The role of toll-like receptors in periodontitis. Oral Dis. 23, 168–180. 10.1111/odi.12468 26923115

[B214] Spiropoulou A. Zareifopoulos N. Bellou A. Spiropoulos K. Tsalikis L. (2019). Review of the association between periodontitis and chronic obstructive pulmonary disease in smokers. Monaldi Arch. Chest Dis. 89, 83–89. 10.4081/monaldi.2019.1018 30968666

[B215] Stein P. S. Steffen M. J. Smith C. Jicha G. Ebersole J. L. Abner E.. (2012). Serum antibodies to periodontal pathogens are a risk factor for Alzheimer's disease. Alzheimer's Dement. 8, 196–203. 10.1016/j.jalz.2011.04.006 22546352 PMC3712346

[B216] Suh J. S. Kim S. Boström K. I. Wang C.-Y. Kim R. H. Park N.-H. (2019). Periodontitis-induced systemic inflammation exacerbates atherosclerosis partly via endothelial-mesenchymal transition in mice. Int. J. Oral Sci. 11, 1–12. 10.1038/s41368-019-0054-1 31257363 PMC6802639

[B217] Sukumar K. Tadepalli A. (2021). Nexus between covid-19 and periodontal disease. J. Int. Med. Res. 49:03000605211002695. 10.1177/03000605211002695 33745336 PMC7989136

[B218] Sulijaya B. Takahashi N. Yamazaki K. (2019). Host modulation therapy using anti-inflammatory and antioxidant agents in periodontitis: a review to a clinical translation. Arch. Oral Biol. 105, 72–80. 10.1016/j.archoralbio.2019.07.002 31288144

[B219] Tan A. Gürbüz N. Özbalci F. İ. Koşkan Ö. Yetkin Ay Z. (2020). Increase in serum and salivary neutrophil gelatinase-associated lipocalin levels with increased periodontal inflammation. J. Appl. Oral Sci. 28:e20200276. 10.1590/1678-7757-2020-0276 32997091 PMC7521419

[B220] Tang Y. Liu J. Zhang D. Xu Z. Ji J. Wen C. (2020). Cytokine storm in covid-19: the current evidence and treatment strategies. Front. Immunol. 11:1708. 10.3389/fimmu.2020.01708 32754163 PMC7365923

[B221] Taskan M. M. Balci Yuce H. Karatas O. Gevrek F. Toker H. (2019). Evaluation of the effect of oleuropein on alveolar bone loss, inflammation, and apoptosis in experimental periodontitis. J. Periodontal Res. 54, 624–632. 10.1111/jre.12662 31032945

[B222] Teixeira F. B. Saito M. T. Matheus F. C. Prediger R. D. Yamada E. S. Maia C. S.. (2017). Periodontitis and Alzheimer's disease: a possible comorbidity between oral chronic inflammatory condition and neuroinflammation. Front. Aging Neurosci. 9:327. 10.3389/fnagi.2017.00327 29085294 PMC5649154

[B223] Torrungruang K. Ongphiphadhanakul B. Jitpakdeebordin S. Sarujikumjornwatana S. (2018). Mediation analysis of systemic inflammation on the association between periodontitis and glycaemic status. J. Clin. Periodontol. 45, 548–556. 10.1111/jcpe.12884 29500831

[B224] Turner M. D. Nedjai B. Hurst T. Pennington D. J. (2014). Cytokines and chemokines: at the crossroads of cell signalling and inflammatory disease. Biochim. Biophys. Acta 1843, 2563–2582. 10.1016/j.bbamcr.2014.05.014 24892271

[B225] Valentine J. Saladyanant T. Ramsey K. Blake J. Morelli T. Southerland J.. (2016). Impact of periodontal intervention on local inflammation, periodontitis, and HIV outcomes. Oral Dis. 22, 87–97. 10.1111/odi.12419 27109277 PMC4899823

[B226] Van Dyke T. E. (2020). Shifting the paradigm from inhibitors of inflammation to resolvers of inflammation in periodontitis. J. Periodontol. 91(Suppl. 1), S19–S25. 10.1002/JPER.20-0088 32441774 PMC8142079

[B227] Wang C.-W. J. McCauley L. K. (2016). Osteoporosis and periodontitis. Curr. Osteop. Rep. 14, 284–291. 10.1007/s11914-016-0330-3 27696284 PMC5654540

[B228] Wang J. Li H. Li B. Gong Q. Chen X. Wang Q. (2016). Co-culture of bone marrow stem cells and macrophages indicates intermediate mechanism between local inflammation and innate immune system in diabetic periodontitis. Exp. Therap. Med. 12, 567–572. 10.3892/etm.2016.3386 27446245 PMC4950830

[B229] Wang R. P.-H. Ho Y.-S. Leung W. K. Goto T. Chang R. C.-C. (2019). Systemic inflammation linking chronic periodontitis to cognitive decline. Brain Behav. Immun. 81, 63–73. 10.1016/j.bbi.2019.07.002 31279681

[B230] Wang Y. Hu Y. Pan K. Li H. Shang S. Wang Y.. (2020). *In-vivo* imaging revealed antigen-directed gingival b10 infiltration in experimental periodontitis. Biochim. Biophys. Acta 1867:165991. 10.1016/j.bbadis.2020.165991 33080346 PMC7722068

[B231] Woelber J. P. Bremer K. Vach K. König D. Hellwig E. Ratka-Krüger P.. (2017). An oral health optimized diet can reduce gingival and periodontal inflammation in humans-a randomized controlled pilot study. BMC Oral Health 17, 1–8. 10.1186/s12903-016-0257-1 27460471 PMC4962497

[B232] Woelber J. P. Tennert C. (2020). Diet and periodontal diseases. Impact Nutr. Diet Oral Health 28, 125–133. 10.1159/000455380 31940617

[B233] Wright D. M. McKenna G. Nugent A. Winning L. Linden G. J. Woodside J. V. (2020). Association between diet and periodontitis: a cross-sectional study of 10,000 nhanes participants. Am. J. Clin. Nutr. 112, 1485–1491. 10.1093/ajcn/nqaa266 33096553

[B234] Xu W. Zhou W. Wang H. Liang S. (2020). Roles of porphyromonas gingivalis and its virulence factors in periodontitis. Adv. Protein Chem. Struct. Biol. 120, 45–84. 10.1016/bs.apcsb.2019.12.001 32085888 PMC8204362

[B235] Yamamoto Y. Saito T. Feng G.-G. Li J. Yasuda Y. Kazaoka Y.. (2016). Intermittent local periodontal inflammation causes endothelial dysfunction of the systemic artery via increased levels of hydrogen peroxide concomitantly with overexpression of superoxide dismutase. Int. J. Cardiol. 222, 901–907. 10.1016/j.ijcard.2016.08.099 27526356

[B236] Ye C. Xia Z. Tang J. Khemwong T. Kapila Y. Kuraji R.. (2020). Unculturable and culturable periodontal-related bacteria are associated with periodontal inflammation during pregnancy and with preterm low birth weight delivery. Sci. Rep. 10, 1–10. 10.1038/s41598-020-72807-9 32978483 PMC7519089

[B237] Yoon Y. Kim T.-J. Lee J.-M. Kim D.-Y. (2018). Sod2 is upregulated in periodontitis to reduce further inflammation progression. Oral Dis. 24, 1572–1580. 10.1111/odi.12933 29972711

[B238] Yoshihara-Hirata C. Yamashiro K. Yamamoto T. Aoyagi H. Ideguchi H. Kawamura M.. (2018). Anti-HMGB1 neutralizing antibody attenuates periodontal inflammation and bone resorption in a murine periodontitis model. Infect. Immun. 86:e00111-18. 10.1128/IAI.00111-18 29531138 PMC5913859

[B239] Yu J. Lin Y. Xiong X. Li K. Yao Z. Dong H. Jiang Z. Yu D. Yeung S.-C. J. Zhang H. (2019). Detection of exosomal PD-L1 RNA in saliva of patients with periodontitis. Front. Genet. 10:202. 10.3389/fgene.2019.00202 30923536 PMC6426748

[B240] Zekeridou A. Mombelli A. Cancela J. Courvoisier D. Giannopoulou C. (2019). Systemic inflammatory burden and local inflammation in periodontitis: what is the link between inflammatory biomarkers in serum and gingival crevicular fluid? Clin. Exp. Dent. Res. 5, 128–135. 10.1002/cre2.162 31049215 PMC6483040

[B241] Zhang Y. Li X. (2015). Lipopolysaccharide-regulated production of bone sialoprotein and interleukin-8 in human periodontal ligament fibroblasts: the role of toll-like receptors 2 and 4 and the MAPK pathway. J. Periodontal Res. 50, 141–151. 10.1111/jre.12193 24854880

[B242] Zhou Z. Liu F. Wang L. Zhu B. Chen Y. Yu Y.. (2020). Inflammation has synergistic effect with nicotine in periodontitis by up-regulating the expression of α7 nachr via phosphorylated gsk-3β. J. Cell. Mol. Med. 24, 2663–2676. 10.1111/jcmm.14986 31930698 PMC7028870

[B243] Zi M. Y. H. Longo P. L. Bueno-Silva B. Mayer M. P. A. (2015). Mechanisms involved in the association between periodontitis and complications in pregnancy. Front. Publ. Health 2:290. 10.3389/fpubh.2014.00290 25688342 PMC4310218

